# Photo-Induced Reactions between Glyoxal and Hydroxylamine in Cryogenic Matrices

**DOI:** 10.3390/molecules27154797

**Published:** 2022-07-27

**Authors:** Barbara Golec, Magdalena Sałdyka, Zofia Mielke

**Affiliations:** 1 Institute of Physical Chemistry, Polish Academy of Sciences, 01-224 Warsaw, Poland; 2 Faculty of Chemistry, University of Wroclaw, 50-383 Wrocław, Poland; zofia.mielke@chem.uni.wroc.pl

**Keywords:** matrix isolation, infrared spectroscopy, photochemistry, carbonyl, hydroxyketene, hemiaminal

## Abstract

In this paper, the photochemistry of glyoxal–hydroxylamine (**Gly–HA**) complexes is studied using FTIR matrix isolation spectroscopy and ab initio calculations. The irradiation of the **Gly–HA** complexes with the filtered output of a mercury lamp (λ > 370 nm) leads to their photoconversion to hydroxyketene–hydroxylamine complexes and the formation of hydroxy(hydroxyamino)acetaldehyde with a hemiaminal structure. The first product is the result of a double hydrogen exchange reaction between the aldehyde group of **Gly** and the amino or hydroxyl group of **HA**. The second product is formed as a result of the addition of the nitrogen atom of **HA** to the carbon atom of one aldehyde group of **Gly**, followed by the migration of the hydrogen atom from the amino group of hydroxylamine to the oxygen atom of the carbonyl group of glyoxal. The identification of the products is confirmed by deuterium substitution and by MP2 calculations of the structures and vibrational spectra of the identified species.

## 1. Introduction

Glyoxal, HCOHCO (**Gly**), the simplest α-dicarbonyl, plays a significant role in atmospheric photochemistry. It is formed in the atmosphere as a product of the oxidation of volatile organic compounds (VOCs) [1], and the direct photolysis of glyoxal is its main removal route from the atmosphere [2,3]. The gas-phase photodissociation of glyoxal involves several fragmentation channels, whose relative contributions depend on the excitation wavelength and other factors such as the occurrence of intermolecular collisions [4,5,6,7,8,9,10]. Moreover, glyoxal is also absorbed by cloud droplets, where it participates in complex photochemical transformations [11,12,13,14,15].Our group has reported systematic study of the photochemical behavior of molecular complexes of glyoxal with atmospherically relevant molecules such as water, methanol and hydrogen peroxide isolated in cryogenic matrices [16,17,18]. For the glyoxal–methanol complex, a very interesting photochemical behavior was observed, namely, under irradiation in the visible range, glyoxal complexed with methanol isomerized to the rather exotic hydroxyketene molecule, H(OH)C=C=O (Equation (1)) [18].

CHOCHO⋯MeOH → H(OH)C=C=O⋯MeOH(1)

This reaction only occurred for the complex with methanol. The complex of glyoxal with H_2_O_2_ shows a different photochemical behavior, in which the hydrogen peroxide molecule undergoes addition to glyoxal [17]. In contrast, the complex of glyoxal with water does not undergo a photochemical isomerization reaction [16]. The complex of methanol with glyoxal derivative, methylglyoxal, shows similar behavior to the glyoxal–methanol complex [19]. Irradiation of this complex leads to the formation of methylhydroxyketene.

An experimental study in argon matrices shed some light on the isomerization reaction of glyoxal [18], and the mechanism of this reaction has also been extensively studied using theoretical methods [20,21]. Irradiation of the complex with deuterium-enriched methanol, CH_3_OD, produced deuterium-enriched ketene, H(OD)C=C=O. This experimental result indicates that the isomerization occurs via a double hydrogen exchange reaction between glyoxal and methanol (or hydrogen and deuterium in the case of CH_3_OD). Recent advanced theoretical studies on the photoisomerization reaction in the CHOCHO⋯MeOH complex have revealed that it follows a complicated mechanism triggered by the excitation of the complex to the lowest single excited electronic state. The mechanism involves two complementary pathways. One of them takes place entirely in the singlet manifold, and the other also involves the lowest triplet state (T_1_) [20,21].

Here, we present the results of a study of the photochemical behavior, under visible irradiation, of the glyoxal complex with hydroxylamine, CHOCHO⋯NH_2_OH (**Gly–HA**). The structures and energetics of CHOCHO⋯NH_2_OH complexes embedded in solid argon and nitrogen have recently been reported [22]. Studies of the CHOCHO⋯NH_2_OH complex provide new, interesting data on hydrogen exchange reactions in glyoxal complexes. They also indicate the existence of an additional reaction channel by which hydroxylamine undergoes addition to glyoxal, forming a product of hemiaminal structure (hydroxy(hydroxyamino)acetaldehyde). In this way, this unique product could be detected and characterized spectroscopically.

Hemiaminals are important intermediate species in the formation of oximes, which are created in a reaction between a carbonyl compound (aldehyde or ketone) and a nucleophile (alkoxyamine or hydroxylamine). This reaction is stepwise: The first step involves the formation of an unstable intermediate hemiaminal, and the next step, usually catalyzed by acids or bases, proceeds via hydrolysis of the hemiaminal and oxime formation. Oxime formation is employed in numerous scientific fields, e.g., chemical biology, organic synthesis and polymer chemistry [23,24,25], as a versatile conjugation strategy. Studies on hemiaminals are relatively rare due to their low stability, but they can be observed under special conditions. For example, hemiaminals have been observed in solutions at low temperatures (−78, −30 °C in THF) [26] or detected with the help of host-guest chemistry as molecules trapped in deep molecular cavities [27] or in a porous coordination network [28]. They have also been identified in a solid-state reaction as an unstable compound [29]. The first stable open-chain hemiaminal, stabilized by an intramolecular hydrogen bond, was obtained in a crystalline form by condensation of di-2-pyridyl ketone with 4-cyclohexyl-3-thioosemicarbazide [30]. A recent study on a hemiaminal presents a synthesis method for aminomethanol, the simplest of hemiaminals, in low-temperature binary ices of methylamine and oxygen. This study demonstrated that aminomethanol can be formed under astrophysical-like conditions [31]. In the case of our work, the elusive hemiaminal formed in the addition reaction was stabilized by the inert environment of the cryogenic matrix.

The products that were formed under irradiation of the **Gly–HA** complexes embedded in cryogenic matrices were identified by FTIR spectroscopy and ab initio calculations. The results of our study are described below.

## 2. Results and Discussion

**Figure 1:**
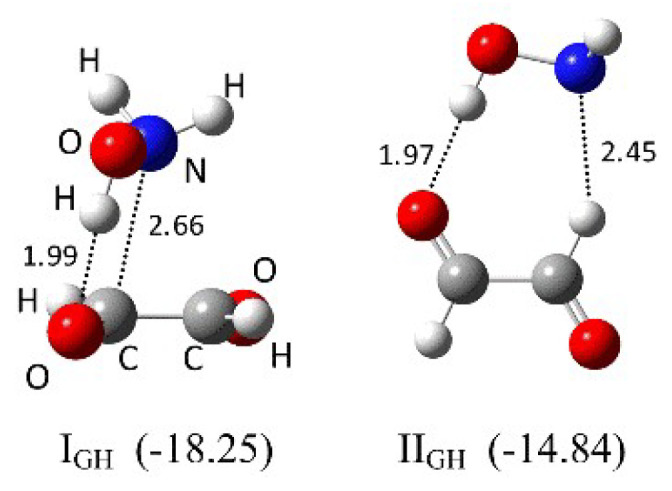
The MP2/6-311++G(2d,2p) optimized structures of the **Gly–HA** complexes identified in argon and nitrogen matrices. The ∆E^CP^_(ZPE)_ binding energies in kJ mol^−1^ are given in parentheses. The intermolecular distances are given in Å.

A study on the interaction of glyoxal with hydroxylamine in solid argon and nitrogen was published recently [22]. The study showed that the non-planar **Gly–HA** complex **I_GH_** was formed in the argon matrix. In the nitrogen matrix, two types of complexes were observed: a non-planar type, **I_GH_**, and a cyclic planar type, **II_GH_** (see Figure 1). The cyclic planar structure of **Gly–HA** complex was more favorable than the non-planar one. The infrared spectra and experimental wavenumbers in comparison with the calculated ones for the CHOCHO⋯NH_2_OH and CHOCHO⋯ND_2_OD complexes are given in the Supplementary Materials (Figure S1 and Table S1).

The UV photochemistry of hydroxylamine has not yet been studied in matrices. Gericke et al. report that the end product of the photolysis of hydroxylamine vapor at 193 nm is H_2_ + HNO [32]. The main dissociation channel leads to H + H + HNO with a quantum efficiency of 1.7 for hydrogen atoms. According to Luckhauset et al., the single-photon irradiation of hydroxylamine at 240 nm produces NH_2_ and OH radicals (mostly in their vibrational ground state) [33]. The photolysis of glyoxal monomers and “cage” dimers in an argon matrix leads to the formation of formaldehyde and carbon monoxide when they are excited in the S_2_ ← S_0_ absorption region (320 nm > λ > 260 nm) [34]. No photoproducts were observed under excitation in the S_1_ ← S_0_ absorption region (λ ~445 nm). Our present study concerning the photolysis of glyoxal in argon and nitrogen matrices showed that only trace amounts of formaldehyde and carbon monoxide were formed when glyoxal was irradiated with a wavelength of λ ≥ 370 nm. Irradiation with the full output of a medium-pressure mercury lamp led to the appearance of a small concentration of carbon dioxide in addition to HCHO and CO. The exposure of the NH_2_OH/Ar(N_2_) matrices to the λ ≥ 370 nm radiation led to the appearance of trace amounts of nitrogen oxide dimers [35] as a result of hydroxylamine degradation, but no HNO was detected [36].

**Figure 2:**
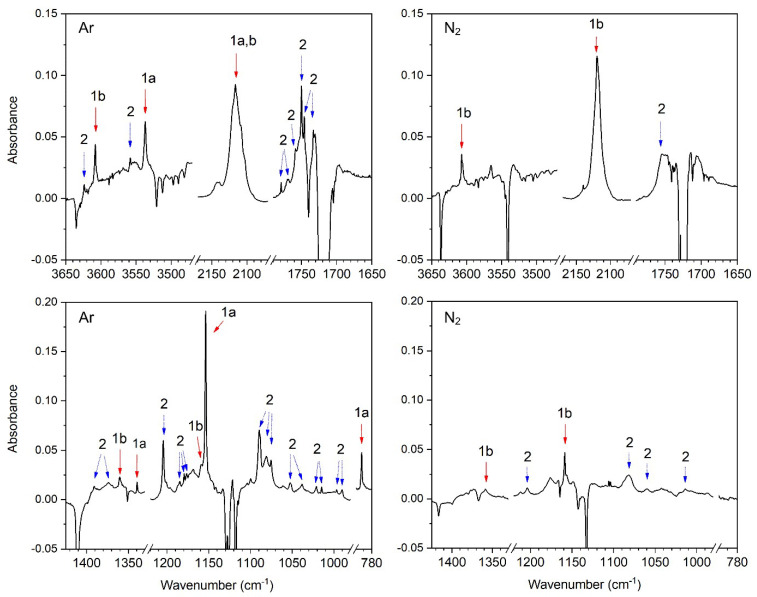
Selected regions of the difference spectrum of the CHOCHO/NH_2_OH/Ar(N_2_) matrix after 90 min of irradiation *minus* freshly deposited CHOCHO/NH_2_OH/Ar(N_2_) matrix at 11 K.

As can be seen in Figure 2, the exposure of **Gly**/**HA**/Ar(N_2_) to irradiation at λ ≥ 370 nm led to a decrease in bands due to the glyoxal–hydroxylamine complexes, and simultaneously a set of new bands appeared. The new bands diminished when the matrices were additionally irradiated with the full output of a medium-pressure mercury lamp. The bands that appeared after irradiation were separated into three groups, 1a, 1b and 2, based on their behavior in all the performed experiments. The wavenumbers of the bands assigned to groups 1a, 1b and 2 are listed in Table 1 and Table 2.

### 2.1. Formation of Hydroxyketene–Hydroxylamine Complexes

The sets of bands belonging to groups 1a and 1b (see Figure 2) involve a very intense band in the 2140–2090 cm^−1^ region that is characteristic of a ketene group, which suggests that the HCOHCO⋯NH_2_OH complex undergoes a similar photoconversion reaction to that of HCOHCO⋯CH_3_OH, during which double hydrogen transfer occurs and then a hydroxyketene–hydroxylamine complex is formed (Equation (2)).

HCOHCO⋯NH_2_OH → H(OH)C=C=O⋯NH_2_OH (2)

For the H(OH)C=C=O⋯CH_3_OH complex, the ν_as_(C=C=O) band was observed at ca. 2105 cm^−1^.

Three new bands assigned to groups 1a and 1b appear in the ν(OH) region, one of which has a close wavenumber to the νOH of **HK** in the H(OH)C=C=O⋯CH_3_OH complex (ca. 3400 cm^−1^) [18]. The other absorptions occur in the vicinity of δNOH, ωNH_2_ of **HA** and δCH + ν_s_CCO(νCO) vibrations of **HK** (see Table 1). These spectroscopic data support the conclusion that during the irradiation of the HCOHCO⋯NH_2_OH complexes, their photoconversion to H(OH)C=C=O⋯NH_2_OH complexes (**HK–HA**) takes place.

The bands assigned to groups 1a and 1b are attributed to two different types of **HK–HA** complexes as discussed below. Bands of group 1a are only identified in an argon matrix, while bands of group 1b are observed in both solid argon and nitrogen.

**Figure 3:**
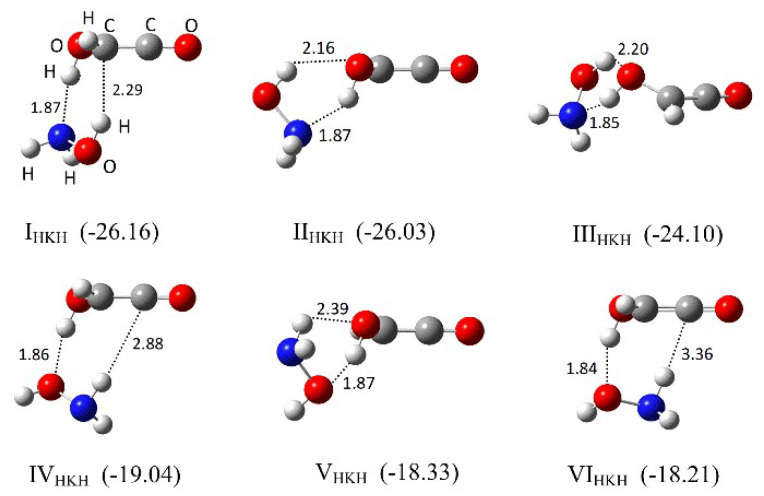
The MP2/6-311++G(2d,2p) optimized structures of the six most stable hydroxyketene–hydroxylamine complexes. The ∆E^CP^_(ZPE)_ binding energies in kJ mol^−1^ are given in parentheses. The intermolecular distances are given in Å.

The structures of **HK–HA** complexes of 1:1 stoichiometry were optimized by the MP2/6-311++G(2d,2p) method. The calculations resulted in twelve stationary points (**I_HKH_–XII_HKH_**), whose structures and ΔE^CP^_(ZPE)_ binding energies are shown in Figure S2 in the Supplementary Materials. The six most stable structures, **I_HKH_–VI_HKH_**, are also presented in Figure 3. The geometrical parameters for these structures are given in Table S2 in the Supplementary Materials. The three most stable structures, **I_HKH_–III_HKH_** (with similar binding energies from −26.16 to −24.10 kJ mol^−1^), are stabilized by the OH…N hydrogen bond between the hydroxyl group of **HK** and the N atom of the amino group of **HA**. Structures **II_HKH_** and **III_HKH_** are additionally stabilized by OH…O interactions, where the hydroxylamine OH group serves as a proton donor and the O atom of the hydroxyl group of hydroxyketene serves as a proton acceptor. The next three structures, **IV_HKH_–VI_HKH_**, are stabilized by the OH…O hydrogen bond that occurs between the hydroxyl group of **HK** and the oxygen atom of the hydroxyl group of **HA**. Complex **V_HKH_** is additionally stabilized by the NH…O interaction of the amino group of **HA** and the hydroxyl group of **HK**. Structures **IV_HKH_–VI_HKH_** are predicted to be about 6–7 kJ mol^−1^ less stable than structures **I_HKH_–III_HKH_**.

In Tables S3–S5 in the Supplementary Materials, the harmonic and anharmonic wavenumbers predicted at the MP2/6-311++G(2d,2p) level for **HA** and **HK** monomers and **HA–HK** complexes (**I_HKH_–VI_HKH_**) are listed. In Table 1, the experimental wavenumbers observed for the photoproducts 1a and 1b are compared with their corresponding calculated wavenumbers of structures **I_HKH_** and **IV_HKH_**, respectively. Because the wavenumbers and intensities of the vibrational bands of complexes **I_HKH_–III_HKH_** are quite similar (see Table S5 in the Supplementary Materials), in Table 1 we only present the data calculated for the most stable structure in the group, **I_HKH_**. The same applies for complexes **IV_HKH_–VI_HKH_**, as in Table 1 we only present the data for the structure **IV_HKH_**. Comparison of the experimental and calculated wavenumbers suggests that the bands of group 1a are due to **HA–HK** complexes stabilized mainly by an OH⋯N bond and represented by structure **I_HKH_**, while the bands assigned to group 1b correspond to complexes stabilized by an OH⋯O bond and represented by structure **IV_HKH_**. The bands of the ν(OH) and τ(OH) vibrations provide the most information on the structure of the complexes. For complex **I_HKH_**, anharmonic MP2 calculations predict intense bands of ν(OH) at 3533 cm^−1^ (calculated intensity I = 365 km mol^−1^) originating from disturbed hydroxylamine as well as ν(OH) at 3260 cm^−1^ (I = 697 km mol^−1^) and τ(OH) at 752 cm^−1^ (I = 119 km mol^−1^) due to perturbed hydroxyketene. In the spectra of the argon matrices, we observed two bands of group 1a at 3537.2 cm^−1^ and 792.2 cm^−1^ corresponding to the disturbed ν(OH) vibration of **HA** and τ(OH) vibration of **HK**, respectively. The ν(OH) absorption of disturbed **HK** was not identified. This is most probably due to the fact that the absorption is broad and diffuse, which is a commonly observed feature for the ν(OH) stretch of the relatively strong O–H⋯O or O–H⋯N bonds [37]. Moreover, possible overlapping of this band with the ν(OH) absorption of the **HA** dimer that occurs in this region [38] makes identification of the band even more difficult. Two additional bands are identified for **I_HKH_** at 1339.6 and 1153.7 cm^−1^. The first one is attributed to the δCH + ν_s_CCO mode of **HK** and the second one to the perturbed ωNH_2_ mode of **HA**, in accordance with the calculations (see Table 1). The calculations predict that for complex **IV_HKH_**, the ν(OH) bands due to disturbed **HA** and **HK** are located at 3667 cm^−1^ (I = 70 km mol^−1^) and 3488 cm^−1^ (I = 548 km mol^−1^), respectively. These bands are identified at 3608.0, 3607.1 cm^−1^ (**HA**) and 3400.0, 3394.5 cm^−1^ (**HK**) in the spectra of the argon and nitrogen matrices, respectively. The ν(OH) band of **HK** in **IV_HKH_** is observed at a very similar wavenumber to the corresponding band of the **HK–Me** complex (3395.7 cm^−1^) [18], which confirms that group 1b belongs to a complex in which **HK** plays the role of proton donor toward the oxygen atom of the OH group of **HA**, forming an O–H⋯O bond (which is the case for structures **IV_HKH_**, **V_HKH_** and **VI_HKH_**).

**Figure 4:**
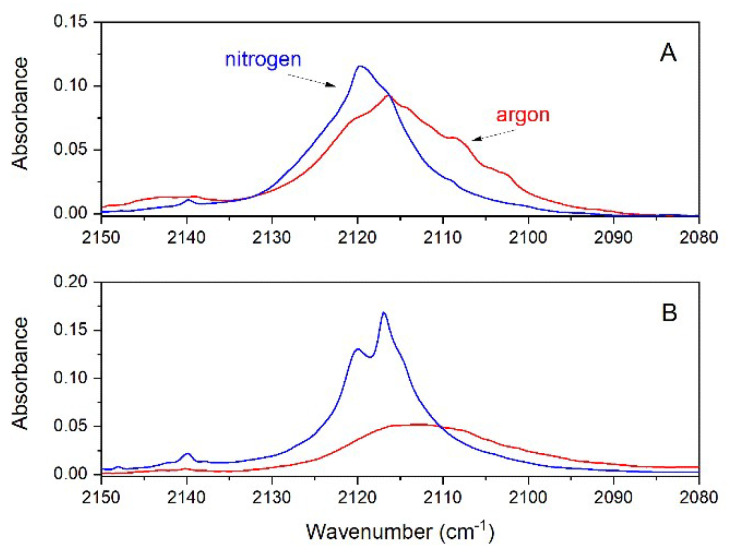
The 2150–2080 cm^−1^ region of the difference spectra of: (**A**) the CHOCHO/NH_2_OH/Ar(N_2_) matrix after 90 min of irradiation *minus* the freshly deposited CHOCHO/NH_2_OH/Ar(N_2_) matrix; (**B**) the CHOCHO/ND_2_OD/Ar(N_2_) matrix after 90 min of irradiation *minus* the freshly deposited CHOCHO/ND_2_OD/Ar(N_2_) matrix.

The shape of the very broad band of the ν_as_C=C=O vibration of **HK** with few subpeaks (or folds) on the band (see Figure 4) suggests that different structures of the **HA–HK** complex with similar wavenumbers of the ν_as_C=C=O mode are probably isolated in the argon and nitrogen matrices. This band is broader in the Ar than in the N_2_ matrix, which is in accordance with the fact that in solid argon all six structures **I_HKH_–VI_HKH_** of **HA–HK** may contribute to its broadness whereas in solid nitrogen it is only the three **IV_HKH_–VI_HKH_**. The MP2 calculations predict that the position of this band in each particular structure differs by a few wavenumbers (from 2114 cm^−1^ to 2123 cm^−1^, see Table S5 in the Supplementary Materials).

**Figure 5:**
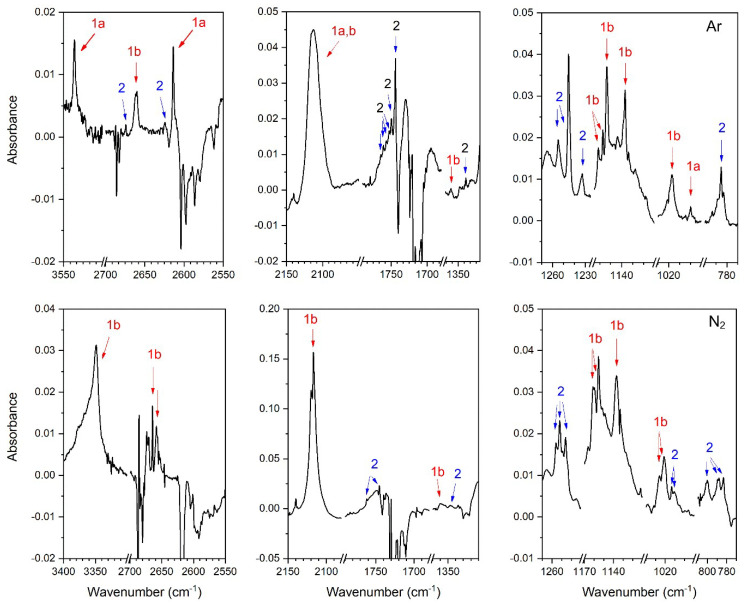
Selected regions of the difference spectrum of the CHOCHO/ND_2_OD/Ar(N_2_) matrix after 90 min of irradiation minus the freshly deposited CHOCHO/ND_2_OD/Ar(N_2_) matrix.

To obtain some information about the mechanism of photoconversion of **Gly–HA** to **HK–HA**, we performed experiments with deuterated hydroxylamine, ND_2_OD (**HA_d_**). The isotopic enrichment was ca. 90% as estimated from the measured and theoretically predicted intensities of the bands due to the ν(OH) and ν(OD) vibrations. The spectra of the matrices doped with **Gly** and **HA_d_** indicate that the complexes **Gly–HA_d_** formed in solid argon and nitrogen have the same structures as those observed for non-deuterated hydroxylamine. The spectra are presented in Figure S1, and the wavenumbers of the bands of **Gly–HA_d_** complexes are listed in Table S1 in the Supplementary Materials. The irradiation (λ > 370 nm) of the **Gly**/**HA_d_**/Ar(N_2_) matrices leads to a decrease in bands due to the **Gly–HA_d_** complexes and due to the formation of the new bands of the photoproducts. Like in the experiments with non-deuterated **Gly–HA** complexes, the bands of photoproducts can be separated into three groups: 1a, 1b and 2. The difference spectra presented in Figure 5 show the bands attributed to all three groups. The identified 1a and 1b bands evidence the formation of the **HK_d_–HA_d_** complexes of hydroxyketene with hydroxylamine, with structures analogical to those observed in the experiment with the non-deuterated **HA** (**I_HKH_** and **IV_HKH_**). The wavenumbers of the identified bands assigned for the **HK_d_–HA_d_** complexes are listed in Table 3.

The spectra recorded after irradiation of the matrices with deuterated hydroxylamine involve numerous bands due to a number of species. The **HK_d_–HA_d_** complexes are formed with relatively low yield, and the identification of their absorptions is quite a difficult task. However, we were able to unequivocally identify in the photolyzed CHOCHO/ND_2_OD/Ar matrices four bands corresponding to group 1a (3536.1, 2614.2, ~2113 and 1000.0 cm^−1^) and eight bands belonging to group 1b (2661.4, ~2113, 1360.4, 1153.6 (subpeaks 1161.2, 1157.2 cm^−1^), 1136.8 and 1017.6 cm^−1^). In the spectra of photolyzed CHOCHO/ND_2_OD/N_2_ matrices, only bands due to group 1b occurred (3349.6, 2664.5, 2495.7, 2116.7, 1360.3, 1160.7, 1155.6, 1137.2 and 1021.4 cm^−1^). The wavenumbers of all bands belonging to groups 1a and 1b are listed in Table 3.

One may expect that the hydrogen and deuterium exchange between glyoxal and deuterated hydroxylamine in the CHOCHO⋯ND_2_OD complexes (both non-planar, **I_GHd_**, and planar, **II_GHd_**, ones) would lead to the formation of H(OD)CCO⋯ND_2_OH and H(OD)CCO⋯NHDOD characterized by similar structures to the non-deuterated complexes **I_HKH_** and **IV_HKH_**. The additional subscript d_4_ or d_2_ is applied to mark whether the complex formed involves ND_2_OH, **I_HKH-d4_**, **IV_HKH-d4_** or NHDOD, **I_HKH-d2_**, **IV_HKH-d2_** (i.e., whether in the exchange process the OD or ND_2_ group of hydroxylamine participates). Among the four identified bands for group 1a in solid argon, the most informative are those observed at 3536.1 and 2614.2 cm^−1^. The first one appears in the OH stretching region of hydroxylamine and shows very good agreement with the wavenumber calculated for complex **I_HKH-d4_** (3533 cm^−1^), which provides strong evidence that in the exchange reaction with the hydrogen atom of CH, the deuterium of the OD group of hydroxylamine participates, leading to the formation of H(OD)CCO⋯ND_2_OH, complex **I_HKH-d4_**. In turn, the wavenumber of the identified 2614.2 cm^−1^ band has practically the same value as that calculated for the OD stretch of NHDOD in complex **I_HKH-d2_** (2615 cm^−1^). The appearance of this band indicates that one of the deuterium atoms of the ND_2_ group participates in the exchange reaction and the H(OD)CCO⋯NHDOD complex **I_HKHd-2_** is formed. The broad, intense ν_as_C=C=O absorption at ca. 2113 cm^−1^ probably involves the corresponding band for both complex **I_HKH-d2_** and complex **I_HKH-d4_**. The band observed at 1000 cm^−1^ is attributed to the δCOD vibration of **I_HKH-d4_** in accordance with calculations, and the corresponding band is also observed for the 1b complex.

Numerous bands were identified for the 1b complexes in both solid argon and nitrogen, and they have close wavenumbers in the spectra of the two matrices. Comparison of the identified wavenumbers with the calculated ones for various types of deuterated complexes shows good agreement with the wavenumbers calculated for complex **IV_HKH-d2_** (Table 3). Strong evidence for **IV_HKH-d2_** formation is provided by the band observed at 2661.4, 2664.5 cm^−1^ in the spectra of solid argon and nitrogen, respectively, which is due to the νOD vibration of perturbed hydroxylamine. Its wavenumber is ca. 45 cm^−1^ higher than the ν(OD) of complex **I_HKH-d2_** (2614.2 cm^−1^), as expected when the OD group of **HA_d_** serves as a proton acceptor. The appearance of this band also shows that in the process of exchange with the H atom of glyoxal, one of the deuterium atoms of the ND_2_ group participates. The bands identified at 3349.6, 1155.6 cm^−1^ (solid nitrogen) and at 1153.6 cm^−1^ (solid argon) are attributed to the νNH and γNHD vibrations according to calculations and are direct evidence for the formation of NHDOD. The other four bands identified for complex **IV_HKH-d2_** are attributed to perturbed **HK_d_** vibrations (see Table 3).

As discussed above, the experiments with ND_2_OD prove that the irradiation of CHOCHO⋯ND_2_OD leads to the formation of H(OD)CCO⋯NHDOD and H(OD)CCO⋯ND_2_OH complexes as bands due to perturbed NHDOD and ND_2_OH molecules can be clearly identified in the spectra. This fact demonstrates that in the hydrogen and deuterium exchange reaction between glyoxal and hydroxylamine, the deuterium atom of the OD group or one of the deuterium atoms of the ND_2_ group participates: (i) CHOCHO⋯ND_2_OD → H(OD)CCO⋯ND_2_OH or (ii) CHOCHO⋯ND_2_OD → H(OD)CCO⋯NHDOD. In the argon matrix, three complexes are probably formed, namely **I_HKH-d2_**, **I_HKH-d4_** and **IV_HKH-d2_**, but in the nitrogen matrix only the latter. Whereas the formation of **IV_HKH-d2_** is very well documented by a number of bands identified for this complex, the presence of **I_HKH-d2_** and **I_HKH-d4_** is evidenced by one band characteristic of the particular complex (νOD **HA_d2_** at 2614.2 cm^−1^ for **I_HKH-d2_** and νOH **HA_d3_** at 3536.1 cm^−1^ for **I_HKH-d4_**). The other two identified bands of group 1a (2113, 1000 cm^−1^) attributed to ν_as_C=C=O and δCOD vibrations may be due to both complex **I_HKH-d2_** and complex **I_HKH-d4_**.

The obtained data show that the non-planar complexes of glyoxal with hydroxylamine, **Gly–HA_d_**, **I_GH_** (stabilized by the OH⋯O bond), that are formed in the argon matrices may photoconvert to the **I_HKH-d2_**, **I_HKH-d4_** and **IV_HKH-d2_ HK_d_–HA_d_** complexes of hydroxyketene with hydroxylamine. In the nitrogen matrices, both the non-planar, **I_GH_**, and planar, **II_GH_** (main product), **Gly–HA_d_** complexes are present (the latter stabilized by OH…O and CH…N bonds), and after conversion the **IV_HKH-d2_ HK_d_–HA_d_** complexes are formed. These data suggest that irradiation of the non-planar **Gly–HA**, **HA_d_** complexes stabilized by the OH…O hydrogen bond, **I_GH_**, leads to double hydrogen exchange (or hydrogen and deuterium exchange) between glyoxal and hydroxylamine in which the hydroxyl or the amino group of **HA**, **HA_d_** may be involved. The irradiation of the planar complex, **II_GH_**, stabilized by OH…O and CH…N hydrogen bonds, proceeds by double hydrogen exchange between the CH group of glyoxal and the amino group of **HA** only.

In the case of the CHOCHO–CH_3_OD complex, the photoconversion resulted in an exchange of proton and deuterium between the CH group of glyoxal and the OD group of methanol, and finally the H(OD)CCO–CH_3_OH complex was formed. According to the coupled-cluster calculations, the non-hydrogen-bonded (non-planar) glyoxal–methanol complex photoconverted initially to a planar hydrogen-bonded complex, which then converted to the final photoproduct (hydroxyketene–methanol complex) [18,20].

### 2.2. Formation of Hydroxy(hydroxyamino)acetaldehyde (Hemiaminal)

**Figure 6:**
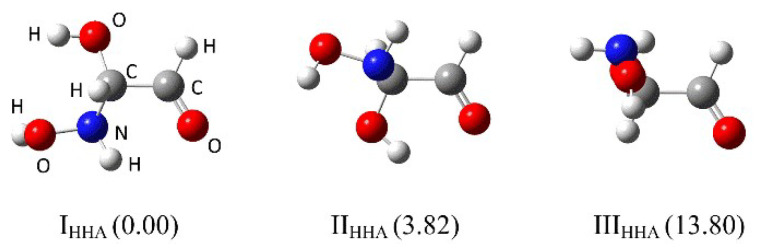
The optimized structures of the three most stable forms of hydroxy(hydroxyamino)acetaldehyde, **HHA**. The computed ∆E_(ZPE)_ energies of **HHA** conformers with respect to the energy of the most stable conformer, **I_HHA_**, in kJ mol^−1^ are given in parentheses.

**Scheme 1:**
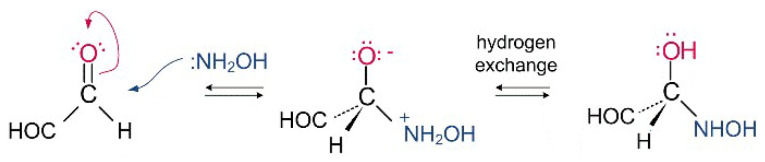
Schematic reaction path for the formation of CHOCH(OH)NHOH, **HHA**, from the CHOCHO–NH_2_OH complex.

Irradiation of **Gly**/**HA**/Ar(N_2_) matrices with a wavelength of λ ≥ 370 nm also leads to the appearance of the group of bands labeled as group 2 (see Figure 2 and Table 4). This group is assigned to different conformers of the product of the addition of hydroxylamine to one of the CHO groups of glyoxal, namely, hydroxy(hydroxyamino)acetaldehyde, **HHA**. The formation of **HHA** proceeds via the addition of the nitrogen atom of the amino group of hydroxylamine to the carbon atom of the carbonyl group of glyoxal and the subsequent migration of the hydrogen atom (from the NH_2_ group of **HA**) to the oxygen atom of the carbonyl group of glyoxal, as presented in Scheme 1. This molecule is formed readily in an argon matrix, but we only observe trace amounts of this photoproduct in a nitrogen matrix. The molecule has not been characterized so far, and its infrared spectrum is unknown. The three most stable structures of this compound predicted at the MP2 level are presented in Figure 6. All forms and their geometrical parameters are given in the Supplementary Materials (Figure S3 and Table S7).

Tables S8 and S9 in the Supplementary Materials show the harmonic and anharmonic wavenumbers and band intensities predicted at the MP2/6-311++G(2d,2p) level and the potential energy distribution (PED) for the two most stable forms of **HHA**. In Table S10, the calculated wavenumbers and band intensities of **I_HHA_–VI_HHA_** structures are given for comparison. In Table 2, the experimental wavenumbers observed for photoproduct **2** are compared with the calculated wavenumbers (and intensities) of the bands predicted for the three most stable conformers of **HHA** (**I_HHA_–III_HHA_**).

The appearance of several bands in the νC=O region and other distinct spectral regions indicates that more than one form of **HHA** is trapped in the argon matrix. The data suggest that one form probably dominates, and another few are isolated in smaller amounts. In the νOH region, we only observe two bands assigned to this group located at 3623.8 and 3558.0 cm^−1^, which, according to the calculations, most suit the **II_HHA_** form. Other identified bands in group 2 also mostly agree with the wavenumbers calculated for the conformer **II_HHA_**. However, we are not able to unequivocally assign the experimental bands to a specific structure because all the structures have similar vibrational characteristics and the amounts of the photoproducts in the matrices are small. Furthermore, the result of the experiment using deuterated hydroxylamine does not give clear evidence as to which conformers of **HHA** are formed in the matrices. The results of this experiment are presented in Figure 5 and Table 2 and in Tables S8, S9 and S11 in the Supplementary Materials.

A photoaddition reaction was also observed as a result of the irradiation (at λ > 345 nm) of the glyoxal–hydrogen peroxide complex in an argon matrix [17], in which 2-hydroxy-2-hydroperoxyethanal was formed. The formation of this photoproduct proceeds via the addition of one of the oxygen atoms of hydrogen peroxide to the carbon atom of the carbonyl group of glyoxal and the subsequent migration of the hydrogen atom (from H_2_O_2_) to the oxygen atom of the carbonyl group of glyoxal.

## 3. Experimental and Computational Methods

Gaseous hydroxylamine (NH_2_OH, **HA**) was prepared from hydroxylamine phosphate salt (95%, Fluka) by heating the salt powder (at 50–65 °C) directly in the deposition line. Deuterated hydroxylamine (ND_2_OD, **HA_d_**) was prepared by heating hydroxylamine phosphate salt in deuterated water (D_2_O) solution and then evaporating off the water under a vacuum. This procedure was repeated several times until the deuteration degree was about 90%. Monomeric glyoxal (CHOCHO, **Gly**) was obtained from solid trimer dihydrate (98%, Sigma) topped with phosphorus pentoxide (P_4_O_10_) powder by heating the materials to 120 °C under a vacuum and then collecting the gaseous glyoxal in a liquid nitrogen trap.

The glyoxal/hydroxylamine/argon (or nitrogen) matrices were prepared by simultaneous deposition of CHOCHO/Ar(N_2_) and NH_2_OH vapor on a cold gold mirror kept at 11–17 K by a closed cycle helium refrigerator (Air Products, Displex 202A). The CHOCHO/Ar(N_2_) matrix concentration was varied in the range of 1/200–1/2000. The absolute concentration of hydroxylamine in the matrices could not be determined, but its concentration was varied by changing the rare gas flow rate and the heating temperature of the hydroxylamine salt. Infrared spectra (4000–600 cm^−1^) with a resolution of 0.5 cm^−1^ were recorded in reflection mode using a Bruker 113v FTIR spectrometer and a liquid N_2_-cooled MCT detector.

The samples deposited in matrices were subjected to the radiation of a 200 W medium-pressure mercury lamp (Philipps CS200W2). A 5 cm water filter (to reduce the infrared light) and a glass long-wavelength pass filter (WG370) were used.

The MP2 method with 6-311++G(2d,2p) basis set was used for geometry optimization of all the monomers and complex structures and calculation of harmonic and anharmonic vibrational spectra of the monomers, complexes and photoproducts [39,40]. Glyoxal under standard conditions exists in a *trans* form [34,41,42], so this conformer was considered exclusively in our study in terms of the nature of its interaction with hydroxylamine. Binding energies were corrected using the Boys–Bernardi full counterpoise procedure [43]. The calculations were performed using the Gaussian 03 program (version C.02) for geometry optimization and Gaussian 16 (version B.01) for frequency calculations [44,45]. The potential energy distribution (PED) of the normal modes was computed using the Gar2ped program [46].

## 4. Conclusions

The photochemistry of glyoxal–hydroxylamine complexes isolated in argon and nitrogen matrices has been investigated using FTIR and MP2/6-311++(2d,2p) calculations. The irradiation of the studied complexes in the visible range (λ > 370 nm) leads to the formation of two types of photoproducts: (i) hydroxyketene–hydroxylamine complexes, **HK–HA**, and (ii) hydroxy(hydroxyamino)acetaldehyde, **HHA**.

The **HK–HA** complexes are formed in the exchange reaction of two hydrogens between two complex subunits, namely, the CH group of **Gly** and the NH or OH group of **HA**. The experiments with deuterated hydroxylamine, ND_2_OD, provide evidence that hydrogen atoms from both the OH and NH_2_ groups of hydroxylamine may participate in the exchange process. The H(OH)CCO…NH_2_OH complexes exist in two forms in the matrices. The first type is stabilized by the OH…N interaction between the hydroxyl group of **HK** and the amino nitrogen of **HA**. In the second type, the OH…O hydrogen bond is formed between the hydroxyl group of **HK** and the oxygen atom of the hydroxyl group of **HA**. These two types of **HK–HA** complex are formed in an argon matrix in noticeable amounts, but in nitrogen only **HK–HA** complexes bonded by the OH…O interaction are created.

Hydroxy(hydroxyamino)acetaldehyde, **HHA**, belonging to the hemiaminals family, is formed by the addition of **HA** to the carbon atom of one aldehyde group of **Gly** and the subsequent migration of the hydrogen atom from the NH_2_ group of **HA** to the oxygen atom of the carbonyl group of **Gly**. In an argon matrix, the **HHA** hemiaminal conformers are observed in noticeable amounts, whereas in a nitrogen matrix the yield of **HHA** is low. The difference in the yield of the products, both **HK–HA** complexes and the **HHA** adduct, in the argon and nitrogen matrices may relate to the fact that different types of glyoxal–hydroxylamine (**Gly–HA**) complexes exist in argon and nitrogen before irradiation.

## Figures and Tables

**Table 1:** Comparison of the observed and calculated anharmonic wavenumbers (cm^−1^) for H(OH)CCO–NH_2_OH.

H(OH)CCO–NH_2_OH					Approximate Description
Exp.	Calc. ^a^	Exp.	Exp.	Calc. ^a^	
1a (Ar)	I_HKH_	1b (Ar)	1b (N_2_)	IV_HKH_	
		3608.0	3607.1	3667 (70)	ν(OH) **HA**, IV_HKH_
3537.2	3533(365)				ν(OH) **HA**, I_HKH_
		3400.0	3394.5	3488 (548)	ν(OH) **HK**, IV_HKH_
	3260 (697)				ν(OH) **HK**, I_HKH_
2116.4 ^b^	2114 (431)	2116.4 ^b^	2119.7	2115 (391)	ν_as_CCO **HK**, I_HKH_
		1360.3	1358.5	1349 (34)	δNOH **HA**, IV_HKH_
1339.6	1353 (47)				δCH + ν_s_CCO **HK**, I_HKH_, IV_HKH_
1153.7	1159 (128) 1154 (26)	1158.6	1158.8	1167 (144) 1126 (43)	ωNH_2_ + δCH **HA**, **HK**, I_HKH_, IV_HKH_
799.8 sh 792.2	752 (119)			623 (102)	τ(OH) **HK**, I_HKH_

^a^ MP2-calculated harmonic intensities (km mol^−1^) are given in parentheses; ^b^ broad band maximum (see in the text).

**Table 2:** Comparison of the observed and calculated anharmonic wavenumbers for CHOCH(OH)NHOH.

CHOCH(OH)NHOH				
Exp. ^a^		Calc. ^b^		
2 (Ar)	2 (N_2_)	I_HHA_	II_HHA_	III_HHA_
3623.8		3676 (63)	3655 (73)	3677 (73)
3558.0		3656 (71)	3578 (71)	3656 (66)
1769.2, 1758.7, **1750.0**, 1745.8, 1733.4	1754.3	1718 (85)	1720 (87)	1721 (92)
1390.9		1393 (7)	1416 (46)	1385 (16)
1373.5		1358 (32)	1394 (12)	1363 (12)
1302.5	1306.3	1293 (29)	1299 (37)	1303 (19)
1228.7, 1226.5, **1204.6**	1203.7	1208 (50)	1249 (29)	1224 (90)
**1185.1**, 1179.4, 1176.5		1169 (55)	1162 (85)	1153 (23)
**1089.3**, 1080.4, 1075.7, 1052.5	**1081.7**, 1060.0	1060 (72)	1036 (29)	1073 (68)
1038.5, **1021.2**, 1014.7	1013.6	1036 (10)	998 (59)	1042 (17)
996.4, **990.2**		984 (94)	953 (14)	990 (59)
**920.8**, 893.1, 887.3, 884.0		916 (34)	871 (68)	951 (3)
835.7, 827.5, **824.0**		806 (36)	806 (31)	884 (66)

^a^ the wavenumber of the most intense line is in bold; ^b^ the MP2-calculated harmonic intensities (km mol^−1^) are given in parentheses.

**Table 3:** Comparison of the observed and calculated anharmonic wavenumbers (cm^−1^) for the H(OD)CCO–ND_2_OH, H(OD)CCO–NHDOD complexes.

H(OD)CCO–NHDOD/ H(OD)CCO–ND_2_OH			H(OD)CCO–NHDOD			Approximate Description
Exp.	Calc. ^a^	Calc. ^a^	Exp. ^b^	Exp. ^b^	Calc. ^a^	
1a (Ar)	I_HKH-d2_	I_HKH-d4_	1b (Ar)	1b (N_2_)	IV _HKH-d2_	
3536.1		3533 (305)				ν(OH) **HA_d4_**, I_HKH-d4_
				3349.6	3356 (43)	ν(NH) **HA_d2_**, IV _HKH-d2_
2614.2	2615 (184)		2661.4	**2664.5 ^c^** 2658.4	2710 (38)	ν(OD) **HA_d2_**, I_HKH-d2,_ IV_HKH-d2_
	2432 (382)	2472 (261) 2410 (148)		2495.7	2579 (278)	ν(OD) **HK_d_**, I _HKH-d2_, I_HKH-d4_, IV_HKH-d2_
2113 ^c^	2114 (441)	2116 (438)	2113 ^c^	2119.6 **2116.7**	2118 (394)	ν_as_CCO **HK**, I_HKH-d2,d4_, IV _HKH-d2_
			1360.4	1360.3	1381 (30)	δCH + ν_s_CCO **HK_d_**, IV_HKH_, IV _HKH-d2_
			1161.2 1157.2 **1153.6**	1160.7 **1155.6**	1179 (32)	γNHD **HA_d2_**, IV _HKH-d2_
			1136.8	1137.2	1169 (25)	δCH + νCO **HK_d_**, IV_HKH-d2_
1000.0	1029 (45)	1028 (49)	1017.6	1026.5 **1021.4**	1009 (53)	δ(COD) **HK_d_**, I_HKH-d2,_ IV _HKH-d2_

^a^ MP2-calculated harmonic intensities (km mol^−1^) are given in parentheses; ^b^ the most intense line of the 2 or 3 lines is in bold; ^c^ broad band maximum (see in the text).

**Table 4:** Comparison of the observed and calculated anharmonic wavenumbers for CHOCH(OD)NDOD.

CHOCH(OD)NDOD				
Exp. ^a^		Calc. ^b^		
2 (Ar)	2 (N_2_)	I_HHA-d_	II_HHA-d_	III_HHA-d_
2674.2		2713 (39)	2702 (40)	2715 (41)
2624.3		2702 (39)	2642 (41)	2702 (40)
1764.2, 1749.6, 1745.4, **1744.0**	1761.9, **1745.0**	1719 (84)	1711 (88)	1721 (90)
1339.3	1346.6	1347 (3)	1367 (12)	1341 (18)
1254.9, **1245.4**, 1233.0	1255.9, **1251.8**, 1246.0	1143 (67)	1176 (40)	1169 (20)
	1094.0	1136 (29)	1101 (29)	1143 (9)
986.2	**1013.3**, 1010.5	990 (8)	974 (41)	992 (2)
788.5, **785.3**, 782.9	800.3, **788.0**, 783.4	750 (58)	729 (35)	745 (43)

^a^ the wavenumber of the most intense line is in bold; ^b^ the MP2-calculated harmonic intensities (km mol^−1^) are given in parentheses.

## Data Availability

The data presented in this study are available in this article and in the Supplementary Materials.

## Supplementary Materials

The following supporting information can be downloaded at: https://www.mdpi.com/article/10.3390/molecules27154797/s1, Table S1: Comparison of the observed wavenumbers (cm^−1^) and wavenumber shifts (Δν = νGH − νM) for the **Gly–HA** (**GH**) complexes present in the Ar and N_2_ matrices with the corresponding calculated values for the complexes **I_GH_** and **II_GH_**. The calculated band intensities are given (in km mol^−1^) in parentheses; Figure S1: Spectra of the CHOCHO/Ar(N_2_) (a), NH_2_OH/Ar(N_2_) (b) and CHOCHO/NH_2_OH(ND_2_OH)/Ar(N_2_) (c) matrices recorded after matrix deposition at 11 K. The bands of **Gly–HA** complexes are indicated by arrows. Dashed and solid arrows correspond to the **I_GH_** and **II_GH_** structures, respectively; Figure S2: The MP2/6-311++G(2d,2p) optimized structures of the hydroxyketene–hydroxylamine (**I_HKH_–XII_HKH_**) complexes. The ∆E^CP^_(ZPE)_ binding energies (in kJ mol^−1^) are given in parentheses. The intermolecular distances are given in Å; Table S2: Selected geometrical parameters of the hydroxylamine and hydroxyketene subunits in their binary complexes (**I_HKH_–VI_HKH_**). For comparison, the corresponding parameters of the monomers (M) are also given. The complexes are numbered in the same way as presented in Figure S2. Bond distances are given in Å, angles in °; Table S3: Calculated harmonic (ν) and anharmonic (ν_anh_) wavenumbers (cm^−1^) and intensities (I, km mol^−1^) for **HA** and **HA_d_**; Table S4: Calculated harmonic (ν) and anharmonic (ν_anh_) wavenumbers (cm^−1^), intensities (I, km mol^−1^) and potential energy distribution (PED) for **HK** and **HK_d_**; Table S5: Calculated harmonic (ν) and anharmonic (ν_anh_) wavenumbers (cm^−1^) and intensities (I, km mol^−1^) for **HK–HA** complexes (**I_HKH_–VI_HKH_**); Table S6: Calculated harmonic (ν) and anharmonic (ν_anh_) wavenumbers (cm^−1^) and intensities (I, km mol^−1^) for deuterated hydroxylamine–hydroxyketene complexes (**I_HKH-d_–VI_HKH-d_**); Figure S3: Optimized structures of hydroxy(hydroxyamino)acetaldehyde (**HHA**). The computed ∆E_(ZPE_) energies of **HHA** conformers with respect to the energy of the most stable conformer, **I_HHA_**, are given in parentheses (in kJ mol^−1^); Table S7: Selected geometrical parameters of the conformers of (hydroxy(hydroxyamino)acetaldehyde (**I_HHA_–XI_HHA_**). The atoms are numbered in the same way as presented in Figure S3. Bond distances are given in Å, angles in °; Table S8: Calculated harmonic (ν) and anharmonic (ν_anh_) wavenumbers (cm^−1^), intensities (I, km mol^−1^) and PED for **I_HHA_** and **I_HHA-d_**; Table S9: Calculated harmonic (ν) and anharmonic (ν_anh_) wavenumbers (cm^−1^), intensities (I, km mol^−1^) and PED for **II_HHA_** and **II_HHA-d_**; Table S10: Calculated harmonic (ν) and anharmonic (ν_anh_) wavenumbers (cm^−1^) and intensities (I, km mol^−1^) for conformers of **HHA** (**I_HHA_–VI_HHA_**); Table S11: Calculated harmonic (ν) and anharmonic (ν_anh_) wavenumbers (cm^−1^) and intensities (I, km mol^−1^) for the deuterated conformers of **HHA** (**I_HHA-d_–VI_HHA-d_**).

## Sample Availability

Samples of the compounds are not available from the authors.
