# Supplementary material for: Photo-Induced Reactions between Glyoxal and Hydroxylamine in Cryogenic Matrices
Source: Molecules. 2022 Jul 27;27(15):4797. doi: 10.3390/molecules27154797 (PMC9369962; doi:10.3390/molecules27154797)
Supplement: Supplementary file 1 [file molecules-27-04797-s001.zip › molecules-1827033-supplementary.pdf]

Supporting Information

Photo-induced reactions between glyoxal and hydroxylamine in cryogenic matrices

Barbara Golec,[a*] Magdalena Sałdyka[b*] and Zofia Mielke[b]

[a] *Institute of Physical Chemistry, Polish Academy of Sciences, Kasprzaka 44/52, 01-224 Warsaw, Poland*
[b] *Faculty of Chemistry, University of Wrocław, F. Joliot-Curie 14, 50-383 Wrocław, Poland*

Corresponding author:
dr Barbara Golec, e-mail: bgolec@ichf.edu.pl
dr Magdalena Sałdyka, e-mail: magdalena.saldyka@chem.uni.wroc.pl

Table S1. The comparison of the observed wavenumbers (cm^{-1}) and wavenumber shifts (Δν = νGH – νM) for the **Gly-HA** (GH) complexes present in the Ar and N_2 matrices with the corresponding calculated values for the complexes **I$_{GH}$** and **II$_{GH}$**. In parentheses the calculated bands intensities are given (km mol^{-1}).

Approximate description	Experimental						Calculated	
	Ar			N_2			Δν	
	ν M	νGH	Δν[1]	ν M	νGH[2]	Δν[1]	I$_{GH}$	II$_{GH}$
**NH_2OH-CHOCHO**								
Hydroxylamine								
**ν(OH)**	3635.5	3521.0<br>3512.4	-118.8	3637.6	*3541.1*<br>3520.9<br>3515.8	*-96.5*<br>-119.2	-145(7)	-106(383)
**δ(NOH)**	1351.2	1412.1<br>1410.2	+60.9	1367.4	*1416.6*<br>1399.7	*+49.2*<br>+32.3	+61(63)	+72(29)
**ω(NH_2)**	1118.3	1129.0<br>1125.8	+9.1	1133.0	*1142.6*	*+9.6*	+14(11)	+24(112)
**ν(NO)**	895.6			895.3	*898.8*	*+3.5*	+14(3)	+9(8)
Glyoxal								
**ν(CH)**	2860.1<br>2854.9	2857.6	-0.4	2857.1	*2875.6*	*+18.5*	-9(60)<br>+12(39)	+15(53)<br>+20(1)
**ν(C=O)**	1724.5	1719.0	-5.5	1730.1	*1720.2*<br>1723.1	*-9.9*<br>-7.0	-4(122)<br>+4(23)	-11(126)<br>-6(20)
**γ(CH)**	812.1<br>807.8			807.4	*820.8*	*+13.4*	+37(0)	+23(8)
**ND_2OD-CHOCHO**								
Hydroxylamine								
**ν(OD)**	2685.1	2604.0<br>2598.0	-84.1	2686.9	*2619.2*<br>*2616.7*	*-68.9*	-106(42)	-77(202)
**δ(NOD)**	1034.5	1068.0<br>1064.5	+33.5	1043.2			+38(6)	+38(7)
**ω(ND_2)**	915.0			916.5	*927.1*	*+10.6*	+0(29)	+14(40)
**ν(NO)**	818.4			827.6	*838.0*<br>*836.6*	*+9.7*	+28(41)	+21(40)
Glyoxal								
**ν(CH)**	2860.1<br>2854.9			2857.1	*2876.3*	*+19.2*	+12(36)<br>-10(59)	+20(1)<br>+15(48)
**ν(C=O)**	1724.5	1719.6	-4.9	1730.1	*1720.5*	*-9.6*	+4(25)<br>-5(125)	-6(20)<br>-11(128)
**δ(CH)**	1313.6	1317.9	+4.3	1322.5			+1(1)<br>+2(4)	+12(5)<br>+11(9)
**γ(CH)**	812.1<br>807.8			807.4	*820.9*	*+13.5*	+41(5)	+22(4)

[1] In the case when the splitting of the band was observed the average of the two wavenumbers at which the two peaks appeared was taken into account to calculate Δν value. [2] The wavenumbers in normal and italic letters are due to structure **I$_{GH}$** and **II$_{GH}$**, respectively.

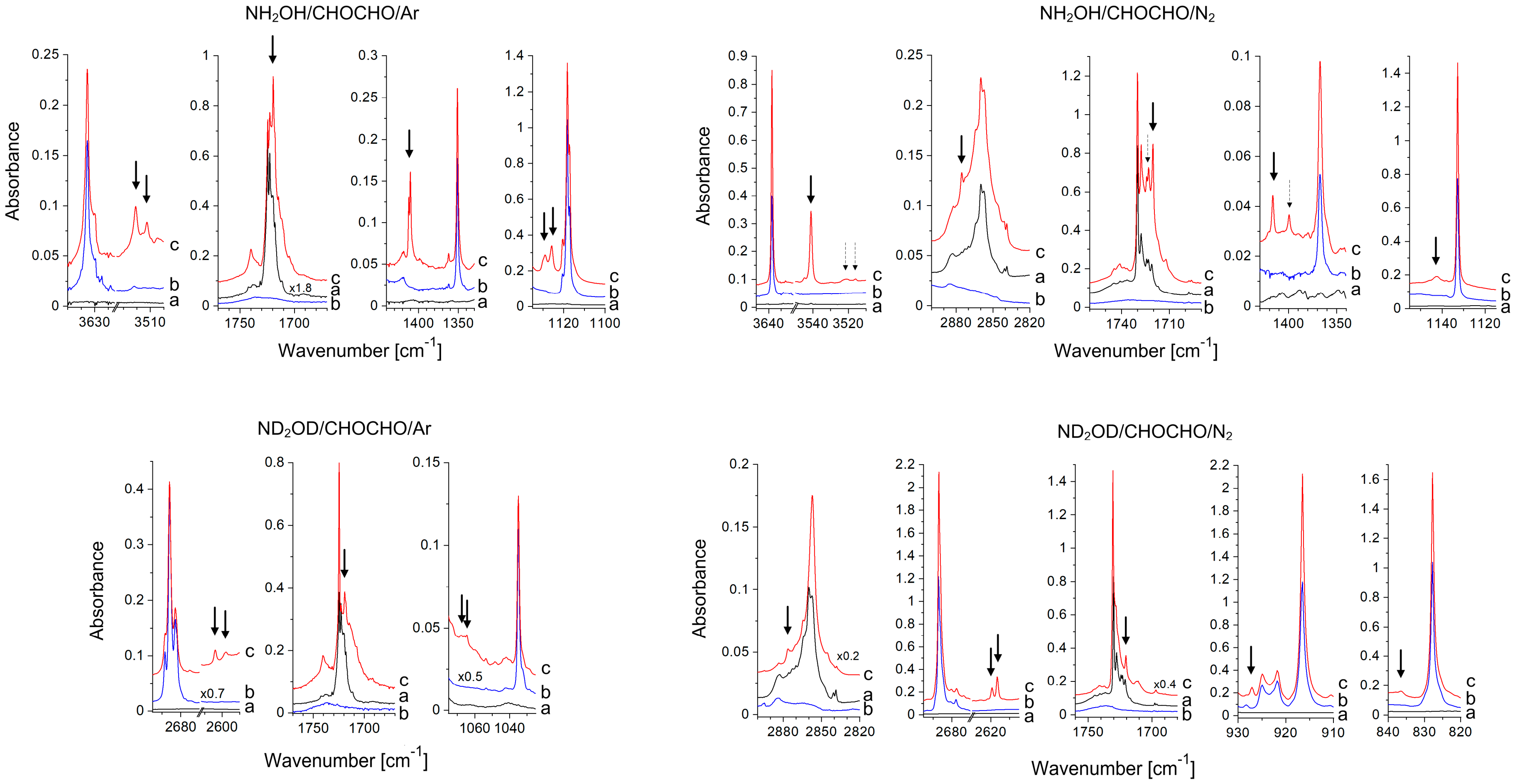

Figure S1. The spectra of the $CHOCHO/Ar(N_2)$ (a), $NH_2OH/Ar(N_2)$ (b) and $CHOCHO/NH_2OH(ND_2OH)/Ar(N_2)$ (c) matrices recorded after matrix deposition at 11 K. The bands of **Gly-HA** complexes are indicated by the arrows. Dashed and solid arrows correspond to the $\mathbf{I_{GH}}$ and $\mathbf{II_{GH}}$ structures, respectively.

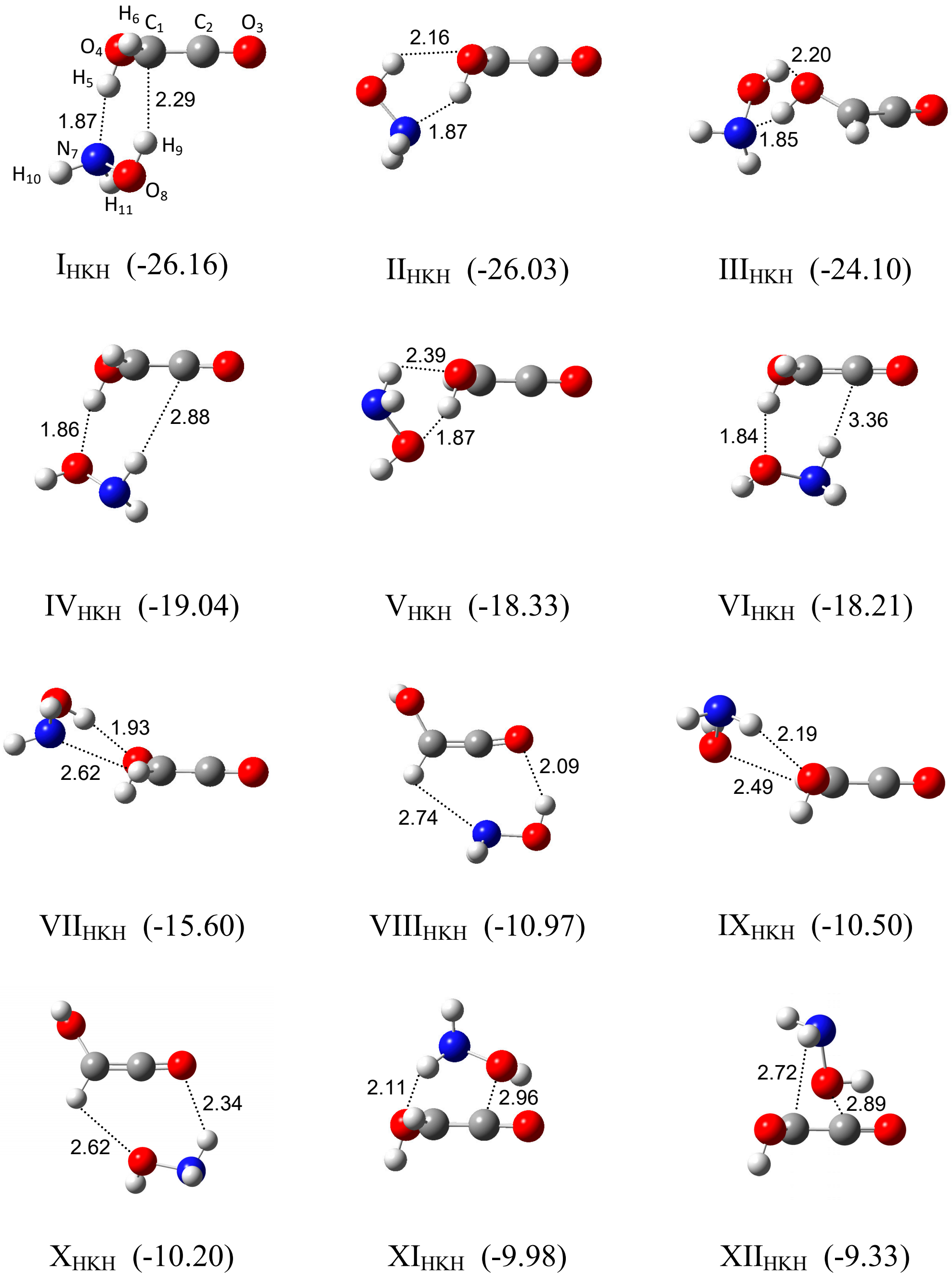

Figure S2. The MP2/6-311++G(2d,2p) optimized structures of the hydroxyketene-hydroxylamine, $\mathbf{I_{HKH}}$ - $\mathbf{XII_{HKH}}$, complexes. The ΔE^{CP}(ZPE) binding energies in kJ mol^{-1} are given in parentheses. The intermolecular distances are given in Å.

Table S2. Selected geometrical parameters of the hydroxylamine and hydroxyketene subunits in their binary complexes (**I_{HKH}-VI_{HKH}**). For comparison the corresponding parameters of the monomers (M) are also given. The complexes are numbered in the same way as presented in Fig. S2. Bond distances are given in Å, angles in °.

Parameter	**M**	**I_{HKH}**	**II_{HKH}**	**III_{HKH}**	**VI_{HKH}**	**V_{HKH}**	**VI_{HKH}**
r C_1-C_2	1.327	1.333	1.327	1.327	1.329	1.327	1.327
r C_2-O_3	1.170	1.170	1.171	1.172	1.171	1.171	1.173
r C_1-O_4	1.398	1.393	1.394	1.394	1.392	1.394	1.393
r C_1-H_6	1.077	1.078	1.078	1.078	1.079	1.078	1.078
r O_4-H_5	0.960	0.978	0.977	0.978	0.970	0.969	0.970
r O_8-H_7	0.959	0.966	0.966	0.965	0.960	0.960	0.960
r O_8-N_9	1.448	1.445	1.443	1.442	1.454	1.455	1.452
r N_9-H_{10}	1.012	1.013	1.012	1.012	1.015	1.015	1.013
r N_9-H_{11}	1.012	1.013	1.012	1.012	1.013	1.012	1.014
R $H_5\cdots N_9$		1.874	1.871	1.853			
R $C_1\cdots H_7$		2.295					
R $H_7\cdots O_4$			2.155	2.198			
R $H_5\cdots O_8$					1.865	1.873	1.842
R $C_1\cdots H_{10}$					2.603		
R $H_{10}\cdots O_4$						2.387	
R $H_{11}\cdots O_4$							2.713
θ C_2-C_1-O_3	179.2	178.7	179.1	179.3	179.2	179.2	179.5
θ C_2-C_1-O_4	119.6	119.6	119.9	120.3	119.6	119.9	119.7
θ C_2-C_1-H_6	119.0	117.8	118.5	118.6	118.2	118.6	118.7
θ H_6-C_1-O_4	120.9	121.0	121.2	120.8	121.3	121.1	121.3
θ C_1-O_4-H_5	107.8	106.3	108.1	107.8	106.8	108.5	107.0
θ H_7-O_8-N_9	101.7	101.8	101.8	101.9	102.2	102.0	102.2
θ O_8-N_9-H_{10}	103.6	104.3	104.4	104.4	103.4	103.5	103.2
θ O_8-N_9-H_{11}	103.6	104.4	104.6	104.5	103.2	103.1	103.7
θ H_{10}-N_9-H_{11}	105.8	106.2	106.4	106.5	106.1	105.8	105.9
θ C_2-C_1-H_7		93.2					
θ O_4-H_5-N_9		162.4	146.0	148.2			
θ H_5-N_9-O_8		104.8	93.1	93.8			
θ H_5-N_9-H_{10}		118.3	122.5	121.8			
θ H_5-N_9-H_{11}		117.3	121.6	121.7			
θ O_4-H_7-O_8			133.0	131.6			
θ O_4-H_5-O_8					164.6	149.0	159.7
θ C_2-C_1-H_{10}					88.1		
θ O_4-H_{10}-N_9						123.9	
θ O_4-H_{11}-N_9							115.4
φ O_3-C_1-C_2-O_4	177.0	-123.2	-160.7	152.6	-161.6	-164.7	132.2
φ O_3-C_1-C_2-H_6	-11.4	71.0	12.9	-21.0	30.0	8.5	-41.3
φ C_2-C_1-O_4-H_5	103.6	103.7	-103.4	101.0	109.5	-102.4	106.9
φ H_6-C_1-O_4-H_5	85.0	-90.9	83.3	-85.6	-81.4	84.6	-79.7
φ H_7-O_8-N_9-H_{10}	-124.8	-125.4	-126.3	-124.5	-122.9	-123.5	-128.6
φ H_7-O_8-N_9-H_{11}	124.8	123.4	122.2	123.9	126.7	126.4	121.1
φ O_4-H_5-N_9-O_8		2.4	3.3	0.0			
φ C_1-O_4-H_5-N_9		-9.8	-120.2	-123.4			
φ H_5-O_4-H_7-O_8			0.0	-0.4			
φ O_4-H_7-N_9-O_8			1.6	0.4			
φ C_1-O_4-H_5-O_8					-23.6	-158.2	-71.2
φ O_4-H_5-O_8-N_9					-6.7	8.0	-18.6
φ C_1-O_4-H_{10}-N_9						90.5	

Table S3. Calculated harmonic (ν) and anharmonic (ν_{anh}) wavenumbers (cm^{-1}) and intensities (*I*, km mol^{-1}) for **HA** and **HA-$_{d}$**.

NH_2OH			ND_2OD			NHDOD			ND_2OH		
**HA**			**HA-$_{d1}$**			**HA-$_{d2}$**			**HA-$_{d3}$**		
ν	ν_{anh}	*I*	ν	ν_{anh}	*I*	ν	ν_{anh}	*I*	ν	ν_{anh}	*I*
3872	3675	54	2821	2716	29	3565	3384	4	3872	3675	54
3609	3422	7	2661	2558	4	2821	2716	29	2661	2558	4
3519	3350	1	2542	2455	0	2600	2504	2	2542	2455	0
1683	1617	18	1219	1184	9	1516	1465	13	1402	1386	43
1416	1361	28	1076	1043	4	1216	1168	32	1217	1182	12
1337	1294	0	987	962	0	1055	1025	6	989	964	0
1163	1124	128	943	912	48	947	917	54	969	936	49
930	894	11	842	819	43	898	868	30	858	836	31
434	407	179	316	301	95	330	314	105	401	378	149

Table S4. Calculated harmonic (ν) and anharmonic (ν_{anh}) wavenumbers (cm^{-1}), intensities (*I*, km mol^{-1}) and potential energy distribution (PED) for **HK** and **HK-d**.

**HK**			
ν	ν_{anh}	*I*	PED[a,b]
3850	3656	71	νOH (99)
3248	3115	18	νCH (99)
2174	2123	379	ν_{as}OCC (99)
1429	1393	12	δCH (48) + ν_sOCC (27) + νC-O (16) + δOH (48)
1285	1242	40	δOH (73) + τCH (27)
1175	1151	71	νC-O (33) + δOH (20) + δCH (19) + ν_sOCC (27)
1027	992	22	νC-O (46) + ν_sOCC (26) + δOCC (23)
682	671	5	δOCC (73) + νC-O (10)
575	580	38	γCH (44) + γOCC (31) + γOH (24)
488	450	44	γOCC (51) + γCH (48)
299	326	115	γOH (74) + γOCC (10) + γCH (10)
226	163	1	δOCC (90?)
**HK-d** (OD)			
3248	3114	18	νCH (99)
2804	2701	39	νOD (100)
2174	2128	379	ν_{as}OCC (99)
1413	1381	30	δCH (56) + ν_sOCC (27) + νC-O (16)
1193	1172	25	δCH (37) + νC-O (33) + ν_sOCC (29)
1026	988	27	νC-O (47) + δOCC (23) + ν_sOCC (25)
955	936	37	δOD (92)
679	666	4	δOCC (74) + νC-O (10)
563	577	28	γCH (45) + γOCC (36) + γOD (16)
485	445	44	γOCC (51) + γCH (48)
246	327	47	δOCC (64) + γOD (31)
205	102	20	γOD (43) + δOCC (42) + γOCC (7)

[a] Frequencies in cm^{-1}, calculated intensities in km mol^{-1}. ν, bond stretching; δ, bending; γ, rocking; τ, torsion; s, symmetric; as, asymmetric.

[b] Potential energy distribution (PED) values greater than 10 % are given.

Table S5. Calculated harmonic (ν) and anharmonic (ν_{anh}) wavenumbers (cm^{-1}) and intensities (I, km mol^{-1}) for **HK-HA** complexes (**I_{HKH}-VI_{HKH}**).

**I_{HKH}**			**II_{HKH}**			**III_{HKH}**			**IV_{HKH}**			**V_{HKH}**			**VI_{HKH}**		
ν	ν_{anh}	I	ν	ν_{anh}	I	ν	ν_{anh}	I	ν	ν_{anh}	I	ν	ν_{anh}	I	ν	ν_{anh}	I
3732	3533	365	3763	3581	*231*	3776	3591	*188*	3863	3667	70	3863	3668	*66*	3862	3668	*69*
3601	3412	15	3606	3418	*15*	3607	3419	*15*	3673	3488	548	3678	3502	*589*	3659	3486	*560*
3517	3351	48	3522	3359	*82*	3521	3350	*16*	3594	3416	25	3597	3468	*14*	3599	3433	*10*
3487	3260	697	3500	3259	*647*	3478	3239	*723*	3496	3328	14	3503	3301	*4*	3506	3330	*0*
3217	3082	22	3237	3101	*28*	3240	3104	*26*	3226	3093	19	3240	3105	*24*	3238	3102	*20*
2161	2114	431	2169	2122	*416*	2165	2118	*375*	2163	2115	391	2169	2123	*405*	2163	2116	*360*
1678	1620	21	1679	1617	*22*	1678	1615	*21*	1685	1621	4	1691	1628	*16*	1688	1623	*15*
1508	1450	4	1506	1444	*39*	1505	1436	*30*	1464	1416	6	1465	1410	*17*	1463	1417	*1*
1468	1407	29	1465	1407	*9*	1467	1400	*25*	1402	1349	34	1405	1362	*38*	1402	1350	*48*
1382	1353	47	1373	1317	*126*	1372	1326	*113*	1361	1319	22	1372	1315	*31*	1355	1313	*1*
1325	1287	0	1322	1284	*3*	1323	1284	*2*	1350	1291	32	1347	1283	*100*	1346	1290	*47*
1192	1159	128	1189	1164	*42*	1189	1165	*52*	1182	1167	144	1189	1178	*24*	1183	1161	*56*
1178	1154	27	1184	1150	*156*	1183	1149	*133*	1178	1126	43	1182	1127	*209*	1174	1125	*142*
1029	996	17	1030	1026	*26*	1030	1033	*24*	1031	993	16	1031	1027	*25*	1031	989	*18*
947	907	9	946	903	*12*	947	904	*11*	925	887	13	922	882	*12*	926	888	*9*
832	752	119	830	720	*189*	842	759	*134*	713	623	102	688	680	*17*	749	616	*119*
688	672	11	685	673	*3*	683	672	*4*	686	677	6	669	590	*93*	685	678	*5*
584	568	64	549	555	*61*	558	551	*72*	559	548	69	536	543	*50*	556	540	*83*
513	515	32	533	445	*78*	509	433	*69*	487	477	15	492	467	*29*	481	469	*23*
482	412	42	481	459	*30*	481	467	*27*	449	401	149	456	376	*141*	457	408	*129*
271	255	3	319	287	*1*	314	280	*1*	235	223	6	246	228	*11*	240	226	*11*
233	219	7	251	231	*9*	256	238	*21*	199	189	5	224	205	*9*	217	200	*2*
217	208	7	227	207	*21*	224	211	*3*	180	167	1	175	79	*2*	151	0	*7*
188	167	0	122	101	*4*	141	118	*2*	132	84	8	123	169	*2*	97	154	*7*
137	119	1	112	104	*7*	93	76	*9*	106	89	1	89	35	*10*	83	95	*3*
53	72	2	36	32	*0*	45	44	*5*	44	41	1	37	31	*0*	27	34	*3*
39	16	9	21	10	*1*	24	17	*0*	38	31	2	11	11	*1*	23	24	*0*

Table S6. Calculated harmonic (ν) and anharmonic (ν_{anh}) wavenumbers (cm^{-1}) and intensities (I, km mol^{-1}) for deuterated hydroxylamine -hydroxyketene complexes (**I_{HKH-d}-VI_{HKH-d}**).

ND_2OD H(OH)CCO			NHDOD H(OD)CCO			NDHOD H(OD)CCO			ND_2OH H(OD)CCO			ND_2OD H(OH)CCO			NHDOD H(OD)CCO			NDHOD H(OD)CCO			ND_2OH H(OD)CCO		
**I_{HKH-d1}**			**$I_{HKH-d2a}$**			**$I_{HKH-d2b}$**			**I_{HKH-d3}**			**II_{HKH-d1}**			**$II_{HKH-d2a}$**			**$II_{HKH-d2b}$**			**II_{HKH-d3}**		
ν	ν_{anh}	*I*	ν	ν_{anh}	*I*	ν	ν_{anh}	*I*	ν	ν_{anh}	*I*	ν	ν_{anh}	*I*	ν	ν_{anh}	*I*	ν	ν_{anh}	*I*	ν	ν_{anh}	*I*
3491	3272	*821*	3558	3375	*9*	3560	3377	*9*	3731	3533	*305*	3506	3273	*809*	3562	3379	*10*	3566	3383	*9*	3761	3580	*167*
3217	3082	*23*	3217	3082	*17*	3217	3082	*17*	3217	3082	*16*	3237	3101	*27*	3237	3101	*20*	3237	3101	*20*	3237	3100	*20*
2719	2614	*138*	2720	2615	*184*	2720	2615	*184*	2655	2551	*8*	2740	2644	*71*	2742	2646	*119*	2742	2645	*119*	2660	2556	*8*
2655	2551	*8*	2597	2498	*4*	2595	2500	*5*	2549	2472	*261*	2660	2556	*8*	2602	2503	*5*	2599	2499	*4*	2559	2417	*335*
2539	2451	*2*	2544	2432	*382*	2544	2431	*381*	2536	2410	*148*	2543	2454	*2*	2554	2437	*379*	2554	2438	*381*	2540	2474	*75*
2160	2113	*434*	2160	2114	*441*	2160	2115	*441*	2160	2116	*438*	2169	2122	*418*	2168	2122	*429*	2168	2122	*428*	2168	2122	*427*
1495	1434	*16*	1508	1462	*13*	1508	1463	*13*	1473	1417	*36*	1474	1436	*12*	1508	1465	*15*	1510	1464	*15*	1485	1437	*67*
1382	1337	*41*	1402	1373	*26*	1402	1373	*25*	1402	1374	*25*	1376	1315	*115*	1417	1387	*39*	1417	1385	*39*	1416	1386	*44*
1215	1183	*9*	1227	1188	*26*	1228	1188	*27*	1214	1182	*9*	1216	1183	*11*	1222	1182	*25*	1227	1186	*27*	1215	1183	*13*
1179	1160	*49*	1192	1170	*23*	1192	1170	*23*	1192	1170	*21*	1190	1166	*62*	1194	1174	*26*	1194	1174	*29*	1194	1174	*27*
1111	1078	*4*	1084	1048	*16*	1080	1044	*10*	1073	1028	*49*	1121	1080	*12*	1098	1064	*23*	1086	1051	*22*	1052	1035	*115*
1029	1002	*19*	1073	1029	*45*	1073	1030	*51*	1027	1010	*11*	1030	986	*21*	1046	991	*109*	1048	1039	*107*	1022	1002	*2*
978	954	*1*	1027	992	*11*	1027	992	*11*	980	957	*0*	977	954	*1*	1022	976	*2*	1022	1003	*2*	979	960	*4*
960	932	*32*	962	941	*34*	964	944	*34*	974	945	*35*	964	935	*35*	965	942	*46*	968	945	*48*	977	943	*39*
874	851	*46*	923	886	*27*	924	886	*25*	888	857	*36*	866	841	*79*	917	881	*36*	917	881	*26*	878	848	*40*
814	740	*89*	685	675	*10*	685	675	*10*	686	674	*13*	822	729	*172*	683	671	*3*	683	670	*3*	683	673	*3*
687	674	*11*	632	600	*70*	632	599	*76*	645	614	*92*	685	672	*4*	621	577	*91*	619	580	*98*	627	578	*108*
575	567	*65*	552	533	*61*	553	533	*55*	550	517	*56*	547	545	*28*	535	510	*27*	535	509	*28*	535	517	*23*
495	475	*35*	493	477	*35*	493	476	*37*	506	537	*26*	487	463	*34*	486	469	*37*	486	469	*38*	517	391	*87*
372	345	*25*	372	347	*21*	372	347	*20*	471	380	*30*	388	341	*56*	392	357	*59*	396	367	*54*	475	507	*32*
229	223	*7*	234	217	*2*	235	234	*5*	228	223	*7*	268	257	*2*	271	249	*0*	284	262	*4*	267	252	*1*
214	202	*6*	227	226	*6*	221	215	*8*	210	202	*2*	233	219	*5*	241	227	*6*	237	230	*5*	236	224	*7*
206	189	*2*	207	197	*6*	206	186	*2*	206	194	*5*	209	187	*18*	220	202	*22*	207	185	*19*	208	188	*20*
176	160	*1*	175	152	*1*	180	158	*0*	176	153	*0*	119	100	*4*	119	100	*4*	119	102	*4*	122	99	*4*
132	118	*1*	132	118	*1*	132	117	*1*	136	118	*1*	105	95	*6*	105	96	*6*	107	97	*7*	104	96	*6*
52	71	*1*	52	74	*1*	53	69	*2*	52	67	*1*	35	38	*0*	36	39	*0*	35	32	*0*	35	40	*0*
36	13	*8*	37	12	*8*	37	18	*8*	36	19	*8*	20	10	*1*	20	9	*1*	20	16	*1*	20	6	*1*

Table S6. Calculated harmonic (ν) and anharmonic ($ν_{anh}$) wavenumbers (cm^{-1}) and intensities (I, km mol^{-1}) for deuterated hydroxylamine -hydroxyketene complexes (**I_{HKH-d}-VI_{HKH-d}**)- continuation.

ND_2OD H(OH)CCO			NHDOD H(OD)CCO			NDHOD H(OD)CCO			ND_2OH H(OD)CCO			ND_2OD H(OH)CCO			NHDOD H(OD)CCO			NDHOD H(OD)CCO			ND_2OH H(OD)CCO		
**III_{HKH-d1}**			**$III_{HKH-d2a}$**			**$III_{HKH-d2b}$**			**III_{HKH-d3}**			**IV_{HKH-d1}**			**$IV_{HKH-d2a}$**			**$IV_{HKH-d2b}$**			**IV_{HKH-d3}**		
ν	$ν_{anh}$	*I*	ν	$ν_{anh}$	*I*	ν	$ν_{anh}$	*I*	ν	$ν_{anh}$	*I*	ν	$ν_{anh}$	*I*	ν	$ν_{anh}$	*I*	ν	$ν_{anh}$	*I*	ν	$ν_{anh}$	*I*
3481	3247	*802*	3565	3382	*10*	3565	3383	*9*	3774	3592	*140*	3673	3486	*533*	3529	3356	*43*	3564	3383	*8*	3863	3667	*77*
3240	3104	*26*	3241	3104	*19*	3241	3104	*19*	3241	3103	*19*	3226	3093	*19*	3226	3093	*16*	3226	3093	*17*	3226	3093	*17*
2749	2652	*58*	2751	2654	*98*	2751	2654	*98*	2660	2556	*8*	2815	2710	*43*	2815	2710	*38*	2815	2710	*38*	2675	2579	*282*
2660	2556	*8*	2601	2502	*5*	2600	2500	*3*	2548	2477	*114*	2650	2549	*17*	2675	2579	*278*	2675	2579	*282*	2650	2549	*12*
2543	2455	*2*	2537	2420	*386*	2537	2420	*390*	2534	2396	*296*	2527	2446	*8*	2600	2505	*2*	2574	2479	*16*	2527	2445	*7*
2164	2118	*374*	2164	2118	*380*	2164	2118	*380*	2164	2118	*381*	2163	2118	*391*	2163	2118	*394*	2162	2118	*395*	2162	2118	*394*
1480	1426	*24*	1508	1463	*16*	1509	1464	*16*	1479	1431	*52*	1463	1418	*8*	1529	1477	*5*	1514	1467	*7*	1412	1381	*27*
1379	1328	*104*	1412	1381	*38*	1412	1381	*38*	1410	1380	*41*	1354	1300	*56*	1412	1381	*30*	1412	1381	*30*	1385	1312	*49*
1216	1182	*11*	1223	1184	*27*	1226	1186	*29*	1215	1181	*13*	1220	1187	*2*	1234	1179	*32*	1223	1177	*31*	1217	1183	*5*
1189	1165	*57*	1195	1172	*28*	1194	1171	*31*	1195	1172	*28*	1181	1156	*55*	1191	1169	*25*	1191	1170	*25*	1191	1169	*26*
1117	1080	*11*	1094	1056	*22*	1087	1050	*16*	1057	1030	*109*	1066	1035	*7*	1053	1021	*7*	1054	1025	*11*	1045	1012	*55*
1030	989	*22*	1049	1010	*99*	1053	1012	*99*	1025	1007	*3*	1031	991	*18*	1045	1009	*53*	1045	1009	*53*	1013	985	*2*
977	954	*0*	1024	1028	*2*	1024	1029	*3*	980	957	*0*	1001	975	*1*	1013	976	*3*	1012	976	*2*	1003	972	*1*
964	933	*33*	966	944	*43*	966	944	*43*	977	949	*40*	941	917	*56*	944	929	*62*	948	931	*65*	968	938	*54*
866	848	*76*	918	882	*32*	917	880	*27*	879	847	*38*	850	818	*38*	900	857	*20*	901	858	*21*	866	842	*24*
834	747	*112*	682	671	*3*	682	673	*3*	682	670	*3*	706	614	*85*	684	673	*5*	684	673	*5*	684	675	*5*
683	670	*3*	634	599	*51*	632	587	*55*	636	591	*61*	686	682	*7*	584	565	*40*	585	566	*41*	587	564	*39*
551	543	*48*	537	518	*63*	536	515	*68*	544	526	*86*	558	543	*63*	504	484	*78*	503	485	*77*	513	479	*127*
485	470	*22*	483	472	*19*	483	472	*19*	488	501	*34*	484	467	*23*	472	428	*27*	472	428	*27*	481	450	*21*
376	333	*50*	380	339	*52*	386	353	*46*	476	376	*41*	334	314	*85*	344	324	*85*	345	325	*88*	401	369	*82*
261	244	*4*	263	237	*2*	279	256	*4*	265	250	*2*	234	225	*5*	232	224	*5*	232	224	*5*	232	224	*6*
240	223	*15*	248	232	*21*	241	224	*17*	240	227	*16*	193	180	*4*	191	177	*4*	190	177	*4*	194	184	*5*
205	189	*3*	216	207	*2*	204	192	*3*	205	188	*4*	151	137	*2*	157	144	*2*	154	123	*1*	151	136	*1*
137	117	*2*	137	115	*2*	137	117	*2*	139	110	*2*	113	98	*4*	118	102	*5*	120	138	*6*	119	114	*6*
87	71	*8*	87	75	*8*	88	73	*8*	87	75	*8*	101	89	*1*	105	88	*1*	102	78	*1*	101	84	*1*
42	50	*4*	43	50	*4*	44	50	*4*	43	50	*4*	43	34	*1*	43	23	*1*	43	23	*1*	43	22	*1*
24	9	*0*	24	10	*0*	24	11	*0*	24	8	*0*	36	29	*1*	36	33	*1*	37	28	*1*	37	33	*2*

Table S6. Calculated harmonic (ν) and anharmonic (ν_{anh}) wavenumbers (cm^{-1}) and intensities (I, km mol^{-1}) for deuterated hydroxylamine -hydroxyketene complexes (**I_{HKH-d}-VI_{HKH-d}**)- continuation.

ND_2OD H(OH)CCO			NHDOD H(OD)CCO			NDHOD H(OD)CCO			ND_2OH H(OD)CCO			ND_2OD H(OH)CCO			NHDOD H(OD)CCO			NDHOD H(OD)CCO			ND_2OH H(OD)CCO		
**V_{HKH-d1}**			**$V_{HKH-d2a}$**			**$V_{HKH-d2b}$**			**V_{HKH-d3}**			**VI_{HKH-d1}**			**$VI_{HKH-d2a}$**			**$VI_{HKH-d2b}$**			**VI_{HKH-d3}**		
ν	ν_{anh}	*I*	ν	ν_{anh}	*I*	ν	ν_{anh}	*I*	ν	ν_{anh}	*I*	ν	ν_{anh}	*I*	ν	ν_{anh}	*I*	ν	ν_{anh}	*I*	ν	ν_{anh}	*I*
3678	3499	*570*	3567	3385	*7*	3537	3367	*26*	3863	3667	*72*	3659	3478	*545*	3566	3385	*7*	3543	3371	*8*	3862	3667	*77*
3240	3105	*24*	3240	3104	*20*	3240	3104	*20*	3240	3104	*20*	3238	3103	*20*	3238	3103	*17*	3238	3103	*17*	3238	3103	*17*
2814	2710	*41*	2814	2710	*36*	2814	2710	*36*	2680	2588	*310*	2814	2710	*45*	2814	2710	*38*	2814	2710	*38*	2665	2574	*288*
2652	2551	*12*	2680	2588	*308*	2680	2588	*303*	2652	2551	*5*	2654	2553	*7*	2665	2574	*292*	2665	2574	*290*	2654	2553	*5*
2532	2453	*4*	2579	2487	*7*	2602	2508	*2*	2532	2452	*3*	2534	2452	*1*	2584	2495	*2*	2601	2506	*3*	2534	2452	*0*
2169	2123	*406*	2168	2123	*410*	2168	2123	*409*	2168	2123	*410*	2163	2116	*360*	2163	2117	*363*	2163	2117	*363*	2163	2117	*362*
1460	1415	*14*	1516	1469	*14*	1539	1486	*16*	1415	1383	*37*	1461	1413	*4*	1520	1471	*12*	1529	1475	*8*	1415	1381	*31*
1364	1302	*150*	1415	1386	*36*	1415	1385	*36*	1383	1345	*39*	1350	1304	*61*	1414	1382	*33*	1414	1381	*32*	1385	1313	*49*
1225	1192	*9*	1227	1182	*34*	1241	1196	*40*	1221	1187	*12*	1222	1188	*8*	1225	1176	*43*	1223	1172	*47*	1219	1184	*12*
1189	1166	*70*	1195	1175	*23*	1195	1174	*23*	1195	1174	*26*	1183	1156	*55*	1191	1168	*29*	1191	1168	*31*	1191	1168	*30*
1065	1034	*8*	1066	1034	*26*	1054	1023	*11*	1040	999	*101*	1066	1035	*6*	1048	1018	*12*	1059	1031	*10*	1043	1006	*52*
1031	1028	*22*	1033	997	*64*	1036	1001	*101*	1021	984	*9*	1031	990	*17*	1043	1004	*47*	1042	1004	*47*	1010	983	*4*
1005	975	*2*	1015	973	*46*	1018	976	*37*	1004	971	*22*	1000	971	*2*	1010	983	*4*	1010	982	*4*	1001	975	*2*
934	921	*57*	944	941	*83*	939	943	*65*	965	956	*72*	939	918	*56*	946	939	*69*	941	935	*61*	966	937	*59*
855	814	*46*	905	843	*20*	901	837	*21*	870	824	*28*	848	814	*39*	903	849	*22*	898	846	*27*	864	835	*27*
687	684	*12*	682	672	*2*	682	671	*2*	682	669	*1*	746	625	*115*	684	674	*4*	684	676	*4*	684	674	*4*
664	575	*93*	571	559	*31*	571	559	*31*	573	554	*32*	685	683	*5*	588	567	*26*	588	566	*26*	589	561	*23*
535	516	*44*	490	473	*40*	490	473	*39*	490	472	*36*	554	539	*65*	511	482	*100*	512	483	*102*	520	473	*135*
485	469	*22*	453	393	*53*	453	392	*52*	476	455	*99*	480	470	*19*	475	437	*12*	475	439	*12*	475	460	*8*
342	309	*91*	348	311	*91*	349	312	*90*	411	306	*78*	340	312	*78*	352	327	*86*	352	323	*84*	423	374	*96*
243	226	*8*	239	227	*8*	239	226	*9*	240	228	*10*	237	224	*10*	235	225	*10*	235	225	*10*	237	224	*10*
220	200	*9*	219	199	*10*	221	203	*10*	220	203	*10*	211	195	*1*	208	199	*2*	209	196	*2*	211	193	*2*
135	84	*1*	150	95	*1*	145	93	*1*	136	85	*1*	123	73	*8*	132	140	*7*	127	130	*6*	126	94	*8*
114	174	*1*	115	167	*1*	118	170	*1*	119	177	*2*	83	156	*3*	86	79	*3*	88	42	*6*	87	-8	*4*
83	33	*10*	84	47	*9*	85	40	*10*	84	21	*9*	79	1	*5*	78	31	*5*	82	87	*3*	79	147	*4*
36	23	*0*	36	12	*0*	36	15	*0*	37	40	*0*	26	56	*2*	27	21	*2*	27	17	*2*	27	46	*2*
10	9	*1*	10	12	*1*	10	11	*1*	10	7	*1*	22	16	*0*	23	39	*0*	22	29	*0*	22	37	*0*

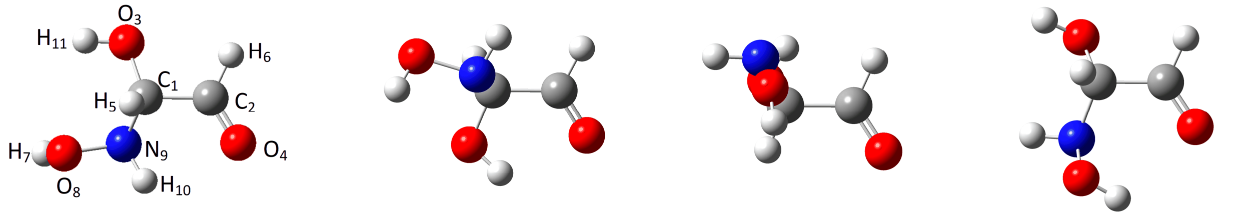

I_{HHA}(0.00) II_{HHA}(3.82) III_{HHA}(13.80) IV_{HHA}(16.14)

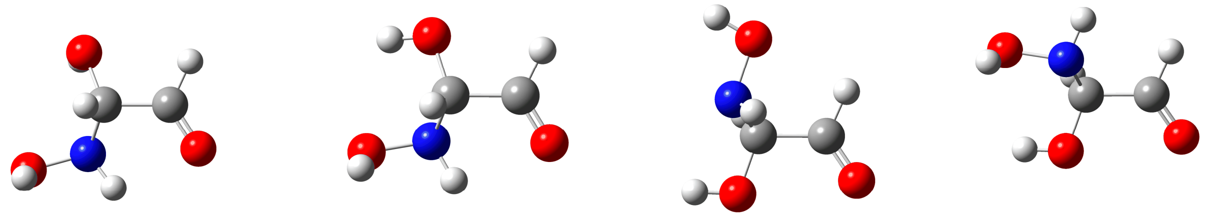

V_{HHA}(17.17) VI_{HHA}(17.46) VII_{HHA}(17.93) VIII_{HHA}(18.65)

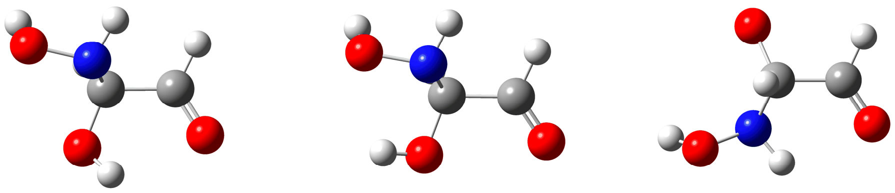

IX_{HHA}(28.52) X_{HHA}(38.55) XI_{HHA}(49.89)

Figure S3. The optimized structures of the hydroxy(hydroxyamino)acetaldehyde, **HHA**. The computed ΔE(ZPE) energies of **HHA** conformers in respect to the energy of the most stable conformer, $\mathbf{I_{HHA}}$, in kJ mol^{-1} are given in parentheses.

Table S7. Selected geometrical parameters of the conformers of (hydroxy(hydroxyamino)acetaldehyde (**I_{HHA}- XI_{HHA}**). The atoms are numbered in the same way as presented in Fig. S3. Bond distances are given in Å, angles in º.

Parameter	**I_{HHA}**	**II_{HHA}**	**III_{HHA}**	**IV_{HHA}**	**V_{HHA}**	**VI_{HHA}**	**VII_{HHA}**	**VIII_{HHA}**	**IX_{HHA}**	**X_{HHA}**	**XI_{HHA}**
r C_1-C_2	1.519	1.522	1.520	1.523	1.530	1.521	1.522	1.520	1.526	1.522	1.526
r C_1-O_3	1.405	1.393	1.419	1.411	1.408	1.405	1.396	1.392	1.388	1.392	1.412
r C_2-O_4	1.214	1.214	1.214	1.212	1.214	1.214	1.212	1.208	1.212	1.207	1.213
r C_1-H_5	1.097	1.093	1.093	1.097	1.098	1.102	1.093	1.099	1.098	1.106	1.092
r C_2-H_6	1.099	1.101	1.102	1.101	1.100	1.099	1.101	1.106	1.102	1.106	1.102
r O_8-H_7	0.961	0.961	0.961	0.963	0.967	0.968	0.961	0.961	0.967	0.968	0.959
r O_8-N_9	1.449	1.442	1.446	1.445	1.426	1.434	1.446	1.449	1.427	1.435	1.439
r N_9-H_{10}	1.016	1.015	1.014	1.013	1.014	1.015	1.016	1.016	1.016	1.015	1.013
r O_3-H_{11}	0.961	0.966	0.961	0.959	0.963	0.962	0.962	0.961	0.965	0.963	0.962
θ C_2-C_1-O_3	107.1	111.4	108.9	106.7	110.9	107.5	108.9	109.7	111.9	110.2	110.6
θ C_1-C_2-O_4	122.1	121.6	122.9	123.7	122.8	121.8	124.3	124.3	121.6	124.3	123.3
θ C_2-C_1-H_5	108.7	110.1	109.4	107.5	107.5	108.2	108.6	107.9	109.2	106.7	108.7
θ O_3-C_1-H_5	110.4	107.6	112.1	111.8	106.0	110.1	84.7	110.8	116.9	110.5	105.6
θ N_9-C_1-H_5	110.6	110.7	112.9	111.1	113.1	111.4	106.0	110.1	111.8	110.7	111.9
θ C_1-C_2-H_6	115.3	117.0	114.5	114.3	114.7	115.4	113.9	114.3	106.3	114.2	114.3
θ O_4-C_2-H_6	122.6	121.4	122.4	122.0	122.5	122.8	121.8	121.3	121.4	121.5	122.4
θ H_7-O_8-N_9	101.9	101.9	102.3	101.1	106.7	106.7	102.1	101.9	106.9	106.8	102.1
θ O_8-N_9-H_{10}	104.7	102.5	104.7	103.1	108.5	108.1	104.1	103.2	106.4	106.7	104.6
θ C_1-N_9-H_{10}	106.8	109.2	107.2	108.0	109.3	108.9	107.7	108.0	109.8	110.2	107.7
θ C_1-O_3-H_{11}	107.4	106.6	107.8	109.1	106.0	107.0	107.0	106.7	107.1	106.5	105.6
φ O_3-C_1-C_2-O_4	-164.9	-7.6	102.2	-138.1	-159.4	-167.9	2.3	-168.8	-12.6	9.3	-151.5
φ O_4-C_2-C_1-H_5	75.8	-126.9	-20.6	101.7	85.1	73.2	-121.8	-108.2	-130.0	-110.8	93.1
φ H_5-C_1-C_2-H_6	-103.5	54.3	163.1	-77.7	-94.1	-106.6	59.7	70.5	51.5	67.6	-86.6
φ N_9-C_1-C_2-H_6	136.7	-65.5	39.2	161.5	143.7	132.9	-56.8	-48.0	-68.7	-51.2	151.6
φ O_3-C_1-C_2-H_6	15.8	173.6	-74.1	42.4	21.2	12.3	-176.2	-168.8	168.9	-172.4	28.8
φ N_9-C_1-O_3-H_{11}	66.6	-95.5	163.2	93.7	-25.8	43.0	56.0	67.9	-81.8	41.2	-42.7
φ O_8-N_9-C_1-H_5	44.9	43.2	-47.6	-39.9	40.7	47.9	-56.0	50.9	48.6	53.1	30.3
φ O_8-N_9-C_1-O_3	-78.9	-76.3	-169.5	-164.3	-78.8	-75.7	-178.2	-72.5	-70.7	-70.1	-87.9
φ H_5-C_1-N_9-H_{10}	-66.2	-67.1	63.3	70.0	-79.5	-69.8	-167.7	-59.0	-68.7	-63.5	-82.5
φ O_3-C_1-N_9-H_{10}	170.0	173.4	-58.7	-54.3	161.0	-75.6	70.2	177.7	171.7	173.3	159.3
φ C_1-N_9-O_8-H_7	130.8	104.8	-128.9	-99.2	-58.5	-62.2	124.1	127.5	-51.1	-59.8	112.1
φ H_7-O_8-N_9-H_{10}	-116.6	-140.2	118.4	147.5	62.2	56.0	-121.9	-119.2	68.3	59.0	-133.1

Table S8. Calculated harmonic (ν) and anharmonic (ν_{anh}) wavenumbers (cm^{-1}), intensities (*I*, $km\ mol^{-1}$) and PED for $\mathbf{I_{HHA}}$ and $\mathbf{I_{HHA\text{-}d}}$.

$\mathbf{I_{HHA}}$			
ν	ν_{anh}	*I*	PED[a,b]
3865	3676	63	ν(C)OH (100)
3854	3656	71	ν(N)OH (100)
3519	3339	16	νNH (100)
3021	2852	52	νCH_{ald} (97)
3017	2911	36	νCH (92)
1751	1718	85	νC=O (85)
1496	1438	5	δNH (55) + δCH (10)
1426	1393	7	δNH (25) + δCH_{ald} (23) + δCH (23)
1400	1358	32	δNOH (30) + δCH (29) + δNH (11)
1397	1364	24	δCH_{ald} (59) + δCOH (11) + δCH (11)
1347	1293	29	δCH (38) + δNOH (37)
1232	1208	50	δCH (47) + δCOH (44)
1208	1169	55	νC-O (35) + γNH (20) + δCCN (16) + νC-N (14)
1093	1060	72	νC-N (34) + γNH (16)
1053	1036	10	γCH_{ald} (31) + νC-C (11) + δCNO (10)
1025	984	94	νC-O (24) + γNH (18) + νC-C (16) + νN-O (16)
953	916	34	νN-O (38) + γNH (23)
826	806	36	νC-N (19) + νN-O (19) + γCH_{ald} (19) + γNH (14)
639	637	25	δCCO (37) + δCCN (16)
556	543	8	τNCO (45) + δCCN (15)
475	470	14	δCCO (33) + δCNO (21) + νC-C (20)
403	381	48	δNOH (68) + δCOH (11)
294	389	50	δCCO (38) + γOH (24)
268	152	116	γ(C)OH (43) + γ(N)OH (24) + δCNO (11)
234	232	9	δCCN (61) + δCCO (14)
127	140	5	τC-C (49) + τN-O (18) + δCNO (11)
87	66	8	τN-O (48) + τC-C (33)

[a] Frequencies in cm^{-1}, calculated intensities in $km\ mol^{-1}$. ν, bond stretching; δ, bending; γ, rocking; τ, torsion; s, symmetric; as, asymmetric.
[b] Potential energy distribution (PED) values greater than 10 % are given.

$\mathbf{I_{HHA\text{-}d}}$			
ν	ν_{anh}	*I*	PED[a,b]
3021	2857	52	νCH_{ald} (97)
3017	2902	35	νCH (97)
2813	2713	39	ν(C)OD (100)
2808	2702	39	ν(N)OD (100)
2573	2478	10	νND (98)
1751	1719	84	νC=O (85)
1427	1397	22	δCH_{ald} (42) + δCH (41)
1382	1347	3	δCH_{ald} (45) + δCH (45)
1367	1325	5	δCH (59) + νC-C (12)
1185	1143	67	νC-N (47) + νC-O (24)
1167	1136	29	νC-O (37) + δND (28)
1120	1094	19	γCH_{ald} (28) + γC-C (15) + δCOD (12) + δCCO (11) + δCH (10)
1042	1015	3	δND (44) + δNOD (25)
1014	990	8	νNO (29) + γCH_{ald} (21) + δNOD (12)
943	913	56	νNO (45) + δNOD (43)
897	884	56	δCOD (59) + γCH_{ald} (12)
830	816	5	δCNO (22) + νC-N (16) + νC-C (11) + γCH_{ald} (11)
776	750	58	γND (49) + δCNO (10) + νNO (10)
618	616	23	γCCO (42) + γCCN (11)
532	515	5	δNCO (45) + δCCN (12) + γND (10)
466	458	10	δCCO (29) + δCNO (23) + νC-C (19)
296	302	34	γOD_{11}(66) + γOD_7(10)
278	262	5	δCCO (43) + δCNO (20) + δCC=O (13)
224	218	6	δCCN (62) + δCC=O (13) + δCNO (10)
202	192	89	γOD_7(63) + γOD_{11}(27)
121	134	4	τC-C (46) + τN-O (23)
85	60	7	τN-O (46) + τC-C (35)

Table S9. Calculated harmonic (ν) and anharmonic ($ν_{anh}$) wavenumbers (cm^{-1}), intensities (*I*, km mol^{-1}) and PED for $\mathbf{II_{HHA}}$ and $\mathbf{II_{HHA\text{-}d}}$.

$\mathbf{II_{HHA}}$			
ν	$ν_{anh}$	*I*	PED[a,b]
3855	3655	73	ν(N)OH (100)
3773	3578	71	ν(C)OH (100)
3522	3344	5	νNH (100)
3065	2927	27	νCH (99)
2993	2838	61	$νCH_{ald}$ (99)
1747	1720	87	νC=O (85)
1516	1469	2	δNH (73) + δNOH (34)
1457	1416	46	δCOH (73) + δCH (34)
1420	1394	12	$δCH_{ald}$ (57) + δCH (19) + δNOH (11)
1376	1330	25	δNOH (33) + $δCH_{ald}$ (18) + δNH (17) + δCOH (13)
1337	1299	37	δCH (50) + δNOH (21)
1297	1249	29	δCH (35) + δCOH (16) + νC-O (16)
1193	1162	85	νC-O (31) + δCH (13) + δCOH (13) + νC-N (11)
1071	1036	29	νC-N (37) + $γCH_{ald}$ (17) + δCCN (11)
1032	998	59	νN-O (31) + $γCH_{ald}$ (12) + δCNO (11)
977	953	14	γNH (30) + νC-C (18) + $γCH_{ald}$ (14) + νC-N (11) + δCCO (11)
905	871	68	γNH (26) + νN-O (25) + νC-O (14) + νC-C (13) + δCNO (11)
831	806	31	γNH (22) + νN-O (17) + νC-C (13) + $γCH_{ald}$ (10) + νC-N (10)
764	742	20	δCC=O (28) + δCC-O (28)
559	546	16	δNCO (62) + γNH (14)
425	433	37	γ(C)OH (19) + δCNO (18) + δCCN (17) + δCC=O (12)
367	329	50	γ(C)OH (58) + δCNO (14)
314	455	85	γ(N)OH (50) + δCNO (18) + δCCN (17)
287	251	42	δCC-O (36) + δCC=O (27)
250	100	21	δNCO (24) + γ(N)OH (22) + γ(C)OH (14) + δCC-O (11)
112	115	11	τCN (61) + γNCO (24)
102	94	2	τC-C (50) + γ(C)OH (35)

[a] Frequencies in cm^{-1}, calculated intensities in km mol^{-1}. ν, bond stretching; δ, bending; γ, rocking; τ, torsion; s, symmetric; as, asymmetric.
[b] Potential energy distribution (PED) values greater than 10 % are given.

$\mathbf{II_{HHA\text{-}d}}$			
ν	$ν_{anh}$	*I*	PED[a,b]
3066	2925	26	νCH (99)
2993	2860	63	$νCH_{ald}$ (98)
2808	2702	40	ν(C)OD (100)
2745	2642	41	ν(N)OD (100)
2576	2484	4	νND (99)
1747	1711	88	νC=O (85)
1417	1388	9	$δCH_{ald}$ (82)
1406	1367	12	δCH (72)
1332	1302	2	δCH (75)
1208	1176	40	νC-O (55)
1143	1101	29	νC-N (40) + δND (38)
1098	1070	7	$δCH_{ald}$ (20) + δNOD (19) + δND (16) + δCOD (16)
1076	1048	12	δNOD (29) + δCOD (14) + νN-O (12) + $γCH_{ald}$ (10)
997	974	41	δCOD (32) + $γCH_{ald}$ (15) + νN-O (12)
944	919	62	δNOD (44) + νN-O (39)
917	892	44	νC-C (29) + νN-O (17) + δCOD (14) + νC-N (12)
846	834	16	$γCH_{ald}$ (19) + νC-N (14) + νC-C (14) + νC-O(13) + δCC=O(12)
739	729	35	γND (47) + δCNO (24) + δCC=O (12)
731	703	21	γND (24) + δCC-O (25) + δCC=O (15)
524	517	14	δNCO (49) + γND (30)
411	403	12	δCNO (27) + δCCN (22) + νC-C (13)
318	328	12	δCC-O (31) + γ(C)OD (25) + δCNO (17) + δCC=O (11)
286	291	19	δCCN (33) + δCNO (22) + τC-C (13) + γ(N)OD (11)
242	227	75	γ(C)OD (39) + δCC-O (20) + γ(N)OD (13)
195	158	21	γ(N)OD (49) + γ(C)OD (16) + δCCN (12)
105	102	9	τC-N (60) + δNCO (12) + τ(N)OD (11)
99	92	3	τC-C (46) + γ(C)OD (38)

Table S10. Calculated harmonic (ν) and anharmonic (ν_{anh}) wavenumbers (cm^{-1}) and intensities (I, km mol^{-1}) for conformers of **HHA (I_{HHA}-VI_{HHA})**.

**I_{HHA}**			**II_{HHA}**			**III_{HHA}**			**IV_{HHA}**			**V_{HHA}**			**VI_{HHA}**		
ν	ν_{anh}	*I*	ν	ν_{anh}	*I*	ν	ν_{anh}	*I*	ν	ν_{anh}	*I*	ν	ν_{anh}	*I*	ν	ν_{anh}	*I*
3865	3676	*63*	3855	3655	*73*	3865	3677	*73*	3885	3695	*66*	3828	3638	*64*	3843	3656	*74*
3854	3656	*71*	3773	3578	*71*	3854	3656	*66*	3828	3628	*71*	3746	3545	*10*	3730	3531	*9*
3519	3339	*16*	3522	3344	*5*	3531	3350	*9*	3546	3364	*5*	3535	3357	*20*	3528	3351	*14*
3021	2852	*52*	3065	2927	*27*	3069	2936	*29*	3022	2896	*40*	3014	2921	*49*	3027	2872	*49*
3017	2911	*36*	2993	2838	*61*	2989	2834	*58*	2996	2837	*64*	3010	2804	*46*	2959	2847	*61*
1751	1718	*85*	1747	1720	*87*	1752	1721	*92*	1758	1735	*79*	1743	1713	*81*	1749	1718	*83*
1496	1438	*5*	1516	1469	*2*	1478	1430	*3*	1515	1463	*4*	1504	1458	*31*	1500	1462	*20*
1426	1393	*7*	1457	1416	*46*	1431	1385	*16*	1448	1397	*9*	1432	1397	*50*	1427	1395	*29*
1400	1358	*32*	1420	1394	*12*	1401	1363	*12*	1414	1381	*10*	1400	1372	*13*	1406	1370	*17*
1397	1364	*24*	1376	1330	*25*	1390	1342	*17*	1368	1326	*18*	1384	1338	*29*	1395	1359	*18*
1347	1293	*29*	1337	1299	*37*	1343	1303	*19*	1326	1283	*68*	1335	1297	*4*	1335	1295	*22*
1232	1208	*50*	1297	1249	*29*	1254	1224	*90*	1242	1212	*83*	1282	1245	*5*	1248	1217	*50*
1208	1169	*55*	1193	1162	*85*	1190	1153	*23*	1171	1136	*1*	1198	1162	*66*	1206	1164	*55*
1093	1060	*72*	1071	1036	*29*	1101	1073	*68*	1123	1089	*58*	1105	1065	*27*	1099	1062	*40*
1053	1036	*10*	1032	998	*59*	1069	1042	*17*	1059	1031	*41*	1065	1044	*22*	1068	1050	*19*
1025	984	*94*	977	953	*14*	1026	990	*59*	1001	974	*58*	1028	990	*53*	1021	980	*55*
953	916	*34*	905	871	*68*	976	951	*3*	934	906	*17*	926	892	*47*	930	896	*33*
826	806	*36*	831	806	*31*	917	884	*66*	834	809	*43*	800	779	*45*	813	784	*52*
639	637	*25*	764	742	*20*	566	568	*5*	642	629	*27*	636	632	*27*	636	638	*21*
556	543	*8*	559	546	*16*	519	504	*2*	520	514	*5*	543	527	*5*	570	548	*5*
475	470	*14*	425	433	*37*	417	507	*132*	425	518	*59*	467	448	*1*	480	482	*11*
403	381	*48*	367	329	*50*	343	229	*13*	376	262	*89*	347	294	*111*	405	361	*140*
294	389	*50*	314	455	*85*	321	318	*5*	339	334	*15*	303	222	*33*	292	427	*23*
268	152	*116*	287	251	*42*	268	27	*92*	261	247	*25*	279	100	*81*	271	38	*78*
234	232	*9*	250	100	*21*	255	461	*32*	235	210	*64*	242	429	*20*	231	211	*6*
127	140	*5*	112	115	*11*	104	118	*4*	140	132	*15*	124	126	*15*	127	143	*16*
87	66	*8*	102	94	*2*	58	34	*12*	112	91	*6*	93	77	*10*	80	57	*8*

Table S11. Calculated harmonic (ν) and anharmonic (ν_{anh}) wavenumbers (cm^{-1}) and intensities (*I*, km mol^{-1}) for the deuterated conformers of **HHA (I_{HHA-d}-VI_{HHA-d})**.

I_{HHA-d}			II_{HHA-d}			III_{HHA-d}			IV_{HHA-d}			V_{HHA-d}			VI_{HHA-d}		
ν	ν_{anh}	*I*	ν	ν_{anh}	*I*	ν	ν_{anh}	*I*	ν	ν_{anh}	*I*	ν	ν_{anh}	*I*	ν	ν_{anh}	*I*
3021	2857	*52*	3066	2925	*26*	3070	2934	*28*	3022	2895	*39*	3014	2920	*45*	3027	2872	*49*
3017	2902	*35*	2993	2860	*63*	2989	2836	*58*	2996	2850	*65*	3010	2807	*47*	2959	2845	*61*
2813	2713	*39*	2808	2702	*40*	2814	2715	*41*	2830	2729	*40*	2785	2685	*41*	2797	2698	*45*
2808	2702	*39*	2745	2642	*41*	2808	2702	*40*	2788	2682	*39*	2729	2622	*5*	2717	2611	*4*
2573	2478	*10*	2576	2484	*4*	2584	2490	*6*	2596	2499	*4*	2587	2497	*12*	2581	2493	*9*
1751	1719	*84*	1747	1711	*88*	1752	1721	*90*	1757	1730	*81*	1742	1712	*81*	1749	1721	*83*
1427	1397	*22*	1417	1388	*9*	1420	1388	*10*	1423	1395	*12*	1410	1378	*28*	1418	1387	*24*
1382	1347	*3*	1406	1367	*12*	1374	1341	*18*	1401	1364	*6*	1368	1328	*6*	1385	1352	*7*
1367	1325	*5*	1332	1302	*2*	1355	1321	*9*	1344	1309	*11*	1353	1319	*3*	1368	1327	*5*
1185	1143	*67*	1208	1176	*40*	1214	1169	*20*	1170	1133	*26*	1199	1160	*16*	1186	1146	*70*
1167	1136	*29*	1143	1101	*29*	1173	1143	*9*	1137	1112	*20*	1176	1142	*74*	1172	1139	*23*
1120	1094	*19*	1098	1070	*7*	1103	1078	*64*	1120	1092	*55*	1112	1083	*7*	1107	1080	*15*
1042	1015	*3*	1076	1048	*12*	1041	1018	*12*	1095	1070	*24*	1069	1038	*18*	1087	1054	*27*
1014	990	*8*	997	974	*41*	1026	992	*2*	1011	983	*2*	1016	986	*12*	1000	976	*11*
943	913	*56*	944	919	*62*	981	959	*20*	936	911	*50*	992	967	*3*	970	950	*1*
897	884	*56*	917	892	*44*	949	919	*48*	904	888	*18*	938	919	*58*	908	887	*67*
830	816	*5*	846	834	*16*	856	844	*29*	854	830	*44*	836	822	*13*	842	830	*11*
776	750	*58*	739	729	*35*	768	745	*43*	752	737	*28*	709	684	*55*	728	702	*54*
618	616	*23*	731	703	*21*	554	536	*6*	605	583	*23*	623	607	*16*	612	609	*11*
532	515	*5*	524	517	*14*	507	495	*4*	511	501	*7*	482	477	*9*	531	512	*1*
466	458	*10*	411	403	*12*	377	377	*64*	381	378	*12*	460	448	*3*	468	460	*5*
296	302	*34*	318	328	*12*	315	321	*1*	334	333	*34*	304	313	*22*	307	271	*76*
278	262	*5*	286	291	*19*	273	294	*35*	305	305	*44*	260	223	*48*	272	320	*14*
224	218	*6*	242	227	*75*	242	204	*8*	234	240	*13*	236	253	*16*	229	225	*7*
202	192	*89*	195	158	*21*	194	174	*62*	179	168	*41*	203	162	*57*	199	96	*56*
121	134	*4*	105	102	*9*	99	112	*3*	129	105	*16*	113	105	*14*	118	150	*12*
85	60	*7*	99	92	*3*	57	38	*11*	111	83	*6*	91	65	*9*	79	47	*7*
